# Gross Primary Productivity of Four European Ecosystems Constrained by Joint CO_2_ and COS Flux Measurements

**DOI:** 10.1029/2019GL082006

**Published:** 2019-05-21

**Authors:** F. M. Spielmann, G. Wohlfahrt, A. Hammerle, F. Kitz, M. Migliavacca, G. Alberti, A. Ibrom, T. S. El‐Madany, K. Gerdel, G. Moreno, O. Kolle, T. Karl, A. Peressotti, G. Delle Vedove

**Affiliations:** ^1^ Department of Ecology University of Innsbruck Innsbruck Austria; ^2^ Department of Biogeochemical Integration Max Planck Institute for Biogeochemistry Jena Germany; ^3^ Department of Agricultural, Food, Environmental and Animal Sciences University of Udine Udine Italy; ^4^ CNR‐IBIMET Firenze Italy; ^5^ Department of Environmental Engineering Technical University of Denmark Kongens Lyngby Denmark; ^6^ INDEHESA‐Forest Research Group Universidad de Extremadura Plasencia Spain; ^7^ Institute of Atmospheric and Cryospheric Sciences University of Innsbruck Innsbruck Austria

**Keywords:** flux partitioning, GPP, LRU, ERU, OCS

## Abstract

Gross primary productivity (GPP), the gross uptake of carbon dioxide (CO_2_) by plant photosynthesis, is the primary driver of the land carbon sink, which presently removes around one quarter of the anthropogenic CO_2_ emissions each year. GPP, however, cannot be measured directly and the resulting uncertainty undermines our ability to project the magnitude of the future land carbon sink. Carbonyl sulfide (COS) has been proposed as an independent proxy for GPP as it diffuses into leaves in a fashion very similar to CO_2_, but in contrast to the latter is generally not emitted. Here we use concurrent ecosystem‐scale flux measurements of CO_2_ and COS at four European biomes for a joint constraint on CO_2_ flux partitioning. The resulting GPP estimates generally agree with classical approaches relying exclusively on CO_2_ fluxes but indicate a systematic underestimation under low light conditions, demonstrating the importance of using multiple approaches for constraining present‐day GPP.

## Key Points

- Traditionally gross primary productivity is inferred from ecosystem‐scale CO_2_ flux measurements
- The proposed joint assimilation of CO_2_ and COS flux measurements avoids the need to specify the leaf relative uptake rate of COS a priori
- The additional information content of ecosystem‐scale COS flux measurements increases inferred gross primary productivity estimates

## 1 Introduction

The net exchange of CO_2_ between an ecosystem and the atmosphere (net ecosystem exchange, NEE) consists of two major components of opposite direction, gross primary productivity (GPP), and ecosystem respiration (Reco). Of these three quantities only NEE can be directly derived at ecosystem level, whereas GPP and Reco have to be inferred from proxies or models (Wohlfahrt & Gu, 2015). For the contemporary carbon cycle, the single most important source for GPP estimates has been NEE measurements by means of the eddy covariance (EC) technique from which GPP as well as Reco are inferred in a standardized fashion by applying so‐called flux partitioning (FP) models (see section 2; Beer et al., 2010; Lasslop et al., 2010; Mahecha et al., 2010; Papale et al., 2006), which exploit the fact that GPP is zero during nighttime and/or depends on solar irradiation during daytime. These FP models, however, have not escaped criticism due to acknowledged problems with some of the underlying data (e.g., potential bias of nighttime EC flux measurements, Aubinet, 2008) and model structural issues (e.g., misrepresentation of sources and drivers of Reco; Heskel et al., 2013; Wehr et al., 2016; Wohlfahrt et al., 2005; Wohlfahrt & Galvagno, 2017), resulting in poorly constrained estimates of uncertainty and the potential for significant bias of the inferred GPP and Reco estimates (Wohlfahrt & Gu, 2015).

In search for further constraints of GPP, the trace gas COS has recently received growing attention (Asaf et al., 2013; Berry et al., 2013; Campbell et al., 2008, 2017; Yang et al., 2018). COS, present in the atmosphere at an average mole fraction of 500 ppt, enters the plant leaf through the stomata in a similar way as CO_2_ where it is catalyzed to hydrogen sulfide (H_2_S) and CO_2_ in a one‐way reaction by the enzyme carbonic anhydrase (CA; Notni et al., 2007; Protoschill‐Krebs & Kesselmeier, 1992). In contrast to CO_2_, whose uptake is always accompanied by release through mitochondrial respiration, the uptake of COS is a one‐way flux (but see Gimeno et al., 2017), opening the opportunity to infer GPP at leaf and canopy scale as (Sandoval‐Soto et al., 2005):

(1) $$\mathrm{GPP}=\left(F_{\mathrm{COS}}\;\chi_{\mathrm{CO}2}\right)/\left(\chi_{\mathrm{COS}}\mathrm{LRU}\right)\;$$

where *F*_COS_ is the COS flux (pmol m^−2^ s^−1^) and *χ*_COS_ (ppt) and *χ*_CO2_ (ppm) are the ambient mole fractions of COS and CO_2_, respectively. Equation (1) is mathematically closed by the so‐called leaf relative uptake rate (LRU) as the ratio of fluxes per unit mole fraction for COS and CO_2_, which must be specified a priori or assessed independently. A recent literature synthesis (Whelan et al., 2018) showed LRU converging to a median of 1.7, but with a wide spread between 0.7 and 6.2 (95% confidence interval of the median). Another critical assumption of applying equation (1) at the ecosystem scale is that nonleaf sources or sinks of COS must be negligible (Wohlfahrt et al., 2012). Previous studies have identified soils to contribute to the ecosystem‐scale COS exchange, either as sinks or sources of COS, even though drivers for differences in direction and magnitude of the soil COS exchange are still poorly understood (Whelan et al., 2018). Previous studies that used COS to estimate ecosystem‐scale GPP relied on a constant, prescribed LRU and neglected any in situ soil contribution to the COS flux (Asaf et al., 2013) or estimated LRU based on in situ branch chamber measurements of COS and CO_2_ (Yang et al., 2018). A third approach made use of a mechanistic ecosystem model to quantify the relationship between COS and CO_2_ fluxes to estimate regional GPP fluxes on the base of airborne COS measurements (Hilton et al., 2017).

This study seeks to address the knowledge gaps in the use of COS as a proxy for ecosystem‐scale GPP and proposes a novel approach for estimating ecosystem‐level GPP based on joint constraints from both CO_2_ and COS fluxes.

## 2 Materials and Methods

### 2.1 Site Description

Field measurements were conducted at four different European biomes in four measurement campaigns: During spring and summer 2015 at an intensively managed temperate mountain grassland (GRA), in spring 2016 at a Mediterranean savanna ecosystem (SAV), in summer 2016 at a temperate beech forest (DBF), and in summer 2017 at an agricultural soybean field (CRO). For further information on all sites, see Table S1 in the suppporting information (Braendholt et al., 2018; El‐Madany et al., 2018; Hörtnagl et al., 2011; Hortnagl & Wohlfahrt, 2014) and Text S1.

### 2.2 Mole Fraction Measurements

The COS and CO_2_ mole fractions were measured using a Quantum Cascade Laser (QCL) Mini Monitor (Aerodyne Research, Billerica, MA, United States) at a wave number of ~2,056 cm^−1^ and at a rate of 5 (SAV, DBF, and CRO) or 10 Hz (GRA). The instrument was placed in a temperature‐controlled box to minimize any influences of ambient temperature changes. The cooling of the QCL and its box was achieved by two Thermocubes (400, Solid State Cooling Systems, Wappinger Falls, NY, United States).

We used valves (Parker‐Hannafin, Cleveland, OH, United States), Teflon™ tubing, stainless steel fittings (SWAGELOK, Solon, OH, United States and FITOK, Offenbach, HE, Germany), and Teflon filters (Savilex, EdenPrarie, MN, United States) to ensure that only materials known not to interact with COS were used for the measurement and calibration airflow. At each field site, we installed the inlet of the intake tube in close proximity to the sonic anemometer. We insulated the tube, which had a diameter of 1/4 inch in GRA and 3/8 inch in the other field sites, and heated it to above ambient temperature to prevent condensation within the tubes. The air was sucked to the QCL at a flowrate of above 7 L/min^−1^ using a vacuum pump (Agilent Technologies, CA, United States).

To correct for the known drift issues of the QCL (Kooijmans et al., 2016), we used a gas with known COS mole fraction to do half hourly calibrations for 1 min. The gas cylinders (working standards) used for the calibrations were either pressurized air (UN 1002), nitrogen (UN 1066), or dried ambient air, which were cross compared (when working standard cylinders were full and close to empty) to an Aculife‐treated aluminum pressurized air cylinder obtained from the National Oceanic and Atmospheric Administration (NOAA). The latter was analyzed by the central calibration laboratory of NOAA for its COS mole fraction using gas chromatography. For additional information see Text S2 (Asaf et al., 2013; Berkelhammer et al., 2014; Campbell et al., 2017; Kooijmans et al., 2016).

### 2.3 Ecosystem Fluxes

The COS and CO_2_ ecosystem fluxes were obtained using the eddy covariance method (Aubinet et al., 1999; Baldocchi, 2014). Besides the fast retrieval of the mole fraction of these two scalars, we used available three‐axis sonic anemometers to obtain high‐resolution data of the three wind components. The list of instruments used in this study is reported in Table S1 (Braendholt et al., 2018; El‐Madany et al., 2018; Hörtnagl et al., 2011; Hortnagl & Wohlfahrt, 2014). The raw data for scalar mole fractions as well as the sonic data were saved on the same PC in SAV and DBF, whereas we used a time synchronization software (NTP, Meinberg, NI, Germany) in GRA and CRO to synchronize two PCs (or a PC and a data logger) saving the data separately. We then used a self‐developed software to determine the time lag, introduced by the separation of tube intake and the sonic anemometer and the tube length, between the mole fraction and sonic dataset (Hortnagl et al., 2010). The data were then processed using the software EdiRe (University of Edinburgh, UK) and MATLAB 2017 (MathWorks, MA, United States). We used laser drift corrected COS‐mole fraction data (see section 2.2) and linear detrending to process our data before following the procedure to correct for sensor response, tube attenuation, path averaging, and sensor separation (Gerdel et al., 2017).

### 2.4 Soil Flux Chamber Measurements

To quantify soil COS fluxes, we installed stainless steel (grade: 316 L) rings 5 cm into the soil, which remained on site for the whole measurement campaign. The aboveground biomass eventually present within each ring was removed at least one day prior to each measurement day, if necessary (GRA, SAV). The vegetation surrounding the rings was allowed to grow and was not cut, the roots within the rings were not removed, and natural litter was left in place. During each measurement, a transparent fused silica glass chamber (Kitz et al., 2017) was placed into a water filled channel of the steel rings, while air was sucked through the chamber to the QCL. We then compared the chamber COS mole fraction with the ambient mole fraction above the chamber, using a second inlet to which we switched before the chamber measurement and after reaching stable readings inside the chamber. The COS soil flux was calculated using the following equation:

(2) $$F=q\left(C_{2}-C_{1}\right)/A$$

where *F* is the COS soil flux (pmol m^−2^ s^−1^), *q* denotes the flowrate in (mol/s), *C*_2_ and *C*_1_ are the chamber and ambient mole fractions of COS in ppt, respectively, and *A* is the soil surface area (0.032 m^2^) covered by the chamber. For a more detailed description see Kitz et al. (2017).

### 2.5 Soil Models

On the basis of the periodically measured soil fluxes and additionally retrieved meteorological and soil data (incident shortwave radiation reaching the soil surface, soil moisture and temperature), a random forest regression model (Liaw & Wiener, 2002) was trained for each site in order to simulate the soil COS exchange at the same time scale as the ecosystem flux measurements (see section 2.3). For additional information on this method see text S3 (Liaw & Wiener, 2002).

### 2.6 Ancillary Data

Standard meteorological parameters and soil related data (e.g., soil temperature and moisture) were measured at each site using state of the art sensors and provided by each site principal investigator (PI) (see Table S1; Braendholt et al., 2018; El‐Madany et al., 2018; Hörtnagl et al., 2011; Hortnagl & Wohlfahrt, 2014).

### 2.7 Flux Partitioning Models

#### 2.7.1 FP Model

Traditionally, GPP on ecosystem level is inferred by applying either a so‐called nighttime (Reichstein et al., 2005) or daytime (Lasslop et al., 2010) FP model. The nighttime FP model makes use of the assumption that the nighttime NEE represents the ecosystem respiration (Reco). Therefore, a Reco model based on a temperature‐dependent function (Lloyd & Taylor, 1994) is fit against the data and used to calculate the daytime respiration.

(3) Reco=rbeE01TRef−T0−1Tair−T0

where Reco denotes the ecosystem respiration (μmol m^−2^ s^−1^), rb is the ecosystem base respiration at the reference temperature *T*_Ref_ (°C), which is set to 15 °C, *T*_air_ (°C) refers to the air temperature (°C), and *E*_0_ (°C) to the temperature sensitivity. *T*_0_ was kept constant at −46.02 °C. GPP can then be retrieved as the difference between the measured NEE and the estimated daytime Reco.

The daytime FP model by Lasslop et al. (2010) uses nighttime data to parameterize the temperature sensitivity (*E*_0_) of Reco via equation (3) but adds a light and temperature dependent function to infer both GPP and Reco from daytime data only:

(4) $$\mathrm{NEE}=\frac{\alpha \;\beta \;R_{\mathrm{PAR}}}{\alpha \;R_{\mathrm{PAR}}+\beta }+\mathrm{rb}\;e^{E_{0}\left(\frac{1}{T_{\mathrm{ref}}-T_{0}}-\frac{1}{T_{\mathrm{air}}-T_{0}}\right)}$$

where *α* denotes the canopy light utilization efficiency (μmol CO_2_/μmol photons), *β* the maximum CO_2_ uptake rate of the canopy at light saturation (μmol CO_2_ m^−2^ s^−1^), and *R*_PAR_ the incoming photosynthetic active radiation (μmol m^−2^ s^−1^). The right‐hand side of the equation, representing ecosystem respiration, follows the same notation as equation (3).

#### 2.7.2 FP+ Model

We extended the FP model to include *F*_COS_ by using the GPP, resulting from the first part on the right‐hand side of equation (4)

(5) $$\mathrm{GPP}\;=\frac{\alpha \;\beta \;R_{\mathrm{PAR}}}{\alpha \;R_{\mathrm{PAR}}+\beta }$$

in equation (6) (rearranged equation (1)):

(6) $$F_{\text{COSmodel}}\;=\;\left(\mathrm{GPP}\;\mathrm{LRU}/\chi_{\mathrm{CO}2}\right)/\chi_{\mathrm{COS}}$$

where *F*_COSmodel_ is the modelled COS flux (pmol m^−2^ s^−1^), *χ*_COS_ (ppt) and *χ*_CO2_ (ppm) are the measured ambient mole fractions of COS and CO_2_, respectively, and LRU (‐) is the leaf relative uptake rate. As COS uptake by CA is thought to be a light‐independent process, while CO_2_ uptake by the enzyme RUBISCO depends on solar radiation absorbed by leaf chlorophyll, LRU was, defined as a light‐dependent parameter, consistent with recent experimental evidence (Kooijmans et al., 2017; Kooijmans et al., 2019; Whelan et al., 2018; Wohlfahrt et al., 2012):

(7) $$\mathrm{LRU}=\iota \;e^{\left(\frac{\kappa }{R_{\mathrm{PAR}}}\right)}$$

Here the parameter *ι* (‐) corresponds to the LRU at high light intensity, the parameter *κ* (μmol m^−2^ s^−1^) governs the increase of LRU at low light conditions, and *R*_PAR_ (μmol m^−2^ s^−1^) represents the incident PAR. In comparison to the FP model, where GPP is obtained by optimization against the measured NEE, in the FP+ model we concurrently optimize equations (4) and (6) against measured NEE and *F*_COS_, GPP thus being derived from two independent constraints.

From the 4–6 unknown model parameters (FP and FP+), we determined the temperature sensitivity parameter of Reco (i.e., *E*_0_) using nighttime data by minimizing the root squared mean error, whereas we used DREAM (Scholz et al., 2017; Vrugt & Ter Braak, 2011), a multichain Markov Chain Monte Carlo algorithm, to infer the remaining 3–5 (see Table S3) parameters based on Bayesian statistics, with daytime data. Preliminary model runs showed that the vapor pressure deficit limitation of GPP (Lasslop et al., 2010) during our field campaigns was minor, so we excluded the parameter controlling this effect from our final model. For additional information on the Bayesian model inversion see Text S4 (Gelman & Rubin, 1992; Schoups & Vrugt, 2010; Van Oijen et al., 2005; Vrugt & Ter Braak, 2011).

## 3 Results and Discussion

**Figure 1:**
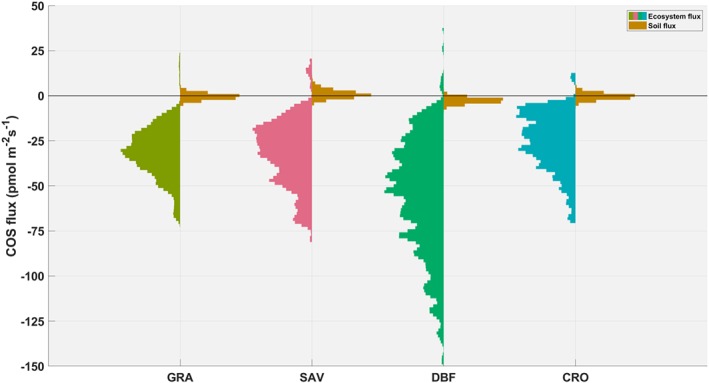
Carbonyl sulfide flux distribution. Distribution plot of the measured daytime carbonyl sulfide (COS) fluxes at ecosystem scale (colored area on the left) and the modeled daytime COS fluxes from soil (brown area on the right) with a bin size of 5 pmol m^−2^ s^−1^ over the course of the campaigns for each ecosystem. Positive fluxes indicate net emission, while negative fluxes indicate net uptake.

Modelled soil COS fluxes ranged from an uptake of −3.57 to an emission of 9.91 pmol m^−2^ s^−1^ with a median of −0.68, 0.67, −2.60, and −0.53 pmol m^−2^ s^−1^ at GRA, SAV, DBF and CRO, respectively (Figure 1). Differences in the sign and magnitude of the soil COS exchange among sites can be explained to a large degree by the magnitude of solar radiation reaching the soil surface (see Text S3), which positively related to the soil COS emission. Sites with a sparse canopy and high amounts of direct solar radiation reaching the soil surface, like SAV, showed stronger COS emission during daytime, whereas during nighttime or at sites with a high leaf area index, uptake was the dominant process for soil COS exchange (Figure 1). Kitz et al. (2017), Whelan and Rhew (2015), and Meredith et al. (2018) suggest that daytime COS emission from soils is mainly linked to abiotic thermal or photodegradation by yet largely unknown reactions, while COS uptake is mostly governed by biological processes, notably the activity of microbial CA (Whelan et al., 2018).

Even though soil COS fluxes overall constituted a small fraction of ecosystem fluxes during daytime (Figure 1), soils, in absolute terms, accounted for up to 10 % (SAV) of the daytime ecosystem COS flux, reaching even higher ratios during dusk and dawn (Figure S4). On the basis of the substantial influence that soils can have on the ecosystem COS exchange during certain times, we corrected the ecosystem COS fluxes for the soil contribution to retrieve the canopy COS uptake.

**Figure 2:**
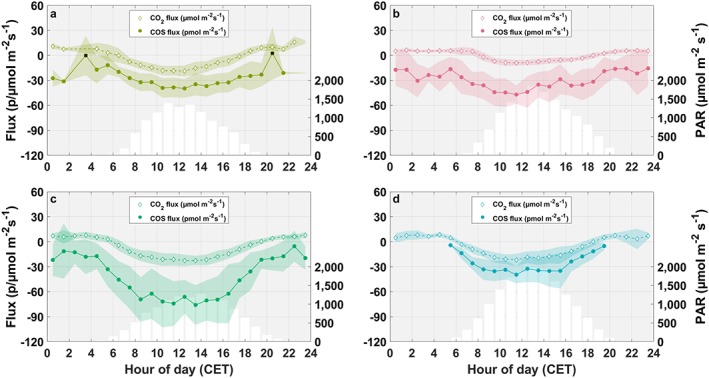
Mean diel carbonyl sulfide and carbon dioxide fluxes. Mean diel variation of the net COS canopy fluxes (filled circles and solid lines) and NEE (open carats and dashed lines) for (a) GRA, (b) SAV, (c) DBF, and (d) CRO over the course of the campaigns. Black xs indicate values below the limit of detection (Langford et al., 2015), which cannot be distinguished from zero fluxes. Shaded areas represent ±1 standard deviation of the mean. Positive fluxes indicate net emission, while negative fluxes indicate net uptake. The photosynthetic active radiation is plotted as hourly means on the right y axis of each plot as a bar graph. COS = carbonyl sulfide; PAR = photosynthetic active radiation; NEE = net ecosystem exchange; CET = Central European Time.

Due to the joint control by stomatal conductance, canopy‐scale COS fluxes and NEE covaried during daytime hours (Figure 2). In contrast to NEE, which turned positive in the absence of photosynthetically active radiation (PAR; i.e. net CO_2_ release), the light‐independent canopy uptake of COS continued at lower rates during night time (Figures 2 and S5–S12) due to incomplete stomatal closure. This finding is in agreement with other studies (Kooijmans et al., 2017, 2019; Novick et al., 2009), although we did not observe an earlier peak in COS uptake as compared to NEE (Figure 2) or GPP as Kooijmans et al. (2019) did. Note that the nighttime residual uptake of COS, when GPP is zero, does not void the general approach of using COS as a proxy for GPP, as this is accounted for by the light‐dependent parameterization of LRU, which approaches infinity at low light (Eq. (7)).

The magnitude of the canopy COS exchange varied strongly between sites, reaching maximum mean uptake rates around 40 pmol m^−2^ s^−1^ (GRA, SAV, and CRO) and up to twice as much at DBF (Figure 2). GRA, DBF, and CRO were characterized by similar maximum mean daytime net CO_2_ uptake rates (~20 μmol m^−2^ s^−1^), whereas SAV exhibited only half of this net uptake (~9 μmol m^−2^ s^−1^; Figure 2). The mean observed ecosystem COS fluxes of our study lie at the upper end or above comparable field observations of the measurements compiled in the recent review by Whelan et al. (2018). The variable canopy COS uptake to NEE ratio (Figures 2 and S13) suggests that either the LRU (equation (1)) must differ between sites, and/or that similar NEE values result from variable GPP to Reco ratios. Unaccounted fluxes of COS within the ecosystems, from stems or sinks and sources of yet unknown origin are another possible but rather unlikely explanation for the differences in the COS uptake to NEE ratio.

**Figure 3:**
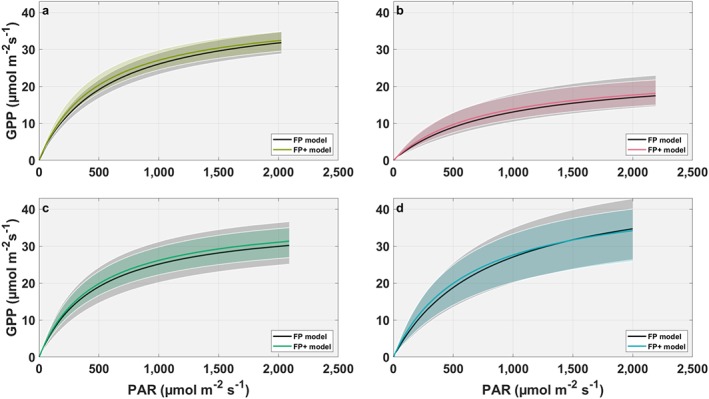
Comparison of model GPP output. GPP (μmol m^−2^ s^−1^) modelled on the basis of the FP (solid black lines) and the FP+ (solid colored lines) model plotted against the measured PAR (μmol m^−2^ s^−1^) for (a) GRA, (b) SAV, (c) DBF, and (d) CRO over the course of the measurement campaigns. Black shaded areas represent the 95% confidence interval of the FP model, whereas the colored shading represents the corresponding 95% confidence interval of the FP+ model. GPP = gross primary productivity; FP = flux partitioning; PAR = photosynthetic active radiation.

GPP resulting from the FP+ model was generally higher than the classical (FP) approach (Figure 3), the difference between the sums of fixed CO_2_ between the models over the course of the measurement campaigns amounting to GRA +5.08% ± 1.23%, SAV +6.08% ± 1.05%, DBF +4.20% ± 0.13%, CRO +1.79% ± 0.74% (the standard deviations representing the temporal variability; see Text S4 and Figures S14–S17). However, model differences were small compared to the model uncertainty calculated from the Bayesian model inversion (see Text S4 and Figure S18).

**Figure 4:**
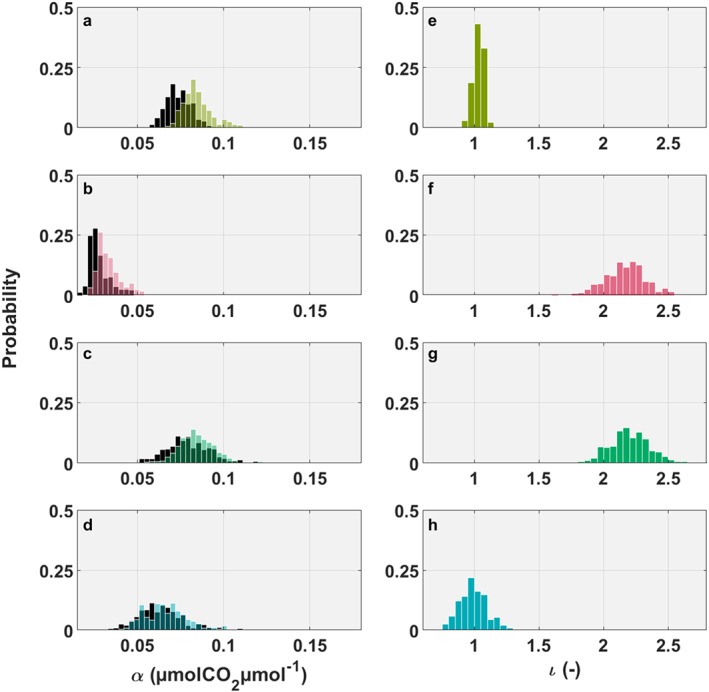
Comparison of model parameter output. Histogram of the probability density function of the last 2,950 runs after convergence of the DREAM algorithm of α, the canopy light utilization efficiency (μmol CO_2_/μmol photons) in the left panels for (a) GRA, (b) SAV, (c) DBF, and (d) CRO and the parameter ι, which is comparable to the LRU at high light conditions in the right panels for (e) GRA, (f) SAV, (g) DBF, and (h) CRO. The FP model is indicated by the black bars, the FP+ model by colored bars. FP = flux partitioning.

The difference between the models is mainly attributable to a higher inferred initial quantum yield (*α*; equation (5)) for all FP+ models (Figure 4), whereas we detected a small decrease in the maximum canopy CO_2_ uptake rate at light saturation (*β*; Figure S19). As a consequence, the absolute difference in GPP between the models for GRA, SAV and DBF increased sharply in the morning, remained relatively stable during the day, and then decreased again in the evening (Figure S20). In contrast to these sites, the FP model predicted higher GPP at higher light conditions for CRO (Figure S20), which caused the absolute model difference in GPP to decline and even reverse sign around noontime (Figure S20). A stepwise regression analysis with the absolute difference in GPP between the FP and FP+ model as dependent variable included PAR at all sites, Tair at GRA, DBF and CRO, soil temperature at GRA, and vapor pressure deficit at neither site.

As hypothesized above, the LRU at saturating light intensity, that is, parameter *ι*, varied strongly across the sites (Figure 4), with the optimal parameter set ranging from 0.89 (CRO) and 1.02 (GRA) for the two herbaceous ecosystems up to 2.22 (DBF) and 2.27 (SAV) for the forest and the mixed woodland grassland site. These values and their mean (1.6) are consistent with the median (1.7) and 95 % confidence interval (0.7 to 6.2) from leaf‐level studies (Whelan et al., 2018). The most productive, that is, highest GPP, ecosystem was CRO, followed by similar GPP at GRA and DBF, and finally SAV (Figures 3 and S19). Not accounting for the soil COS exchange would have resulted in an overestimation of GPP by 1.3%–2.9 % for GRA and DBF and up to 5.7%–8.6 % for CRO and SAV, which cautions against neglecting the soil contribution at sites where solar radiation significantly penetrates to the soil surface. Results were not sensitive to the chosen prior distribution for the parameter *ι*—using a uniform prior distribution would only change the resulting ι by 0.01 (‐) in GRA to up to +0.03 (‐) in DBF (see Table S4), which decreased the GPP of DBF by 1.4 %. In contrast to our results, a recent study using isotopic flux partitioning (FPiso) by Wehr et al. (2016) reported that traditional FP methods (Lasslop et al., 2010; Reichstein et al., 2005) overestimate Reco, which the authors ascribed to the Kok effect (Heskel et al., 2013), and thus in turn GPP. As the FP+ and FPiso models have a quite different theoretical basis, these conflicting results are difficult to reconcile and most likely require joint flux measurements of COS and the isotopologues of CO_2_ to be resolved.

## 4 Conclusions

During recent years COS has seen increasing use as an alternative means of inferring GPP on spatial scales from ecosystem to global (Asaf et al., 2013; Berry et al., 2013; Campbell et al., 2008; Campbell, Berry, et al., 2017; Yang et al., 2018) . The Achilles heel of these promising efforts is the need to specify the LRU a priori (Wohlfahrt et al., 2012), because its variability is not well understood, and the poorly quantified contribution of soils (Whelan et al., 2018). Our study is the first to overcome these issues by treating the LRU as an adjustable parameter, which is jointly optimized against both CO_2_ and COS flux measurements, and explicitly accounts for the soil COS exchange. Although GPP inferred in this fashion agreed well with the one derived from conventional CO_2_ flux partitioning, our FP+ model yielded a slightly higher GPP (by 4.3% ± 1.8%) on average over the course of the measurement campaigns and across all sites. Even though our study indicates a larger uptake of CO_2_ across multiple biomes compared to conventional CO_2_ flux partitioning, our GPP estimate lies within the uncertainty of the GPP reported in Beer et al. (2010) and thus does not support recent reports of substantially higher estimates (Arneth et al., 2017; Welp et al., 2011). To take advantage of newly emerging constraints on GPP, for example, COS (Wohlfahrt et al., 2012), isotopic flux partitioning (Wehr et al., 2016), and Sun‐induced fluorescence (Wohlfahrt et al., 2018), should be compared and combined with traditional flux partitioning to understand the differences between methods and to decrease the overall uncertainty of GPP.

## Supporting information

Supporting Information S1
