# Supplementary material for: Gross Primary Productivity of Four European Ecosystems Constrained by Joint CO2 and COS Flux Measurements
Source: Geophys Res Lett. 2019 May 21;46(10):5284–93. doi: 10.1029/2019GL082006 (PMC6686783; doi:10.1029/2019GL082006)
Supplement: Supplementary file 1 — Supporting Information S1 [file GRL-46-5284-s001.docx]

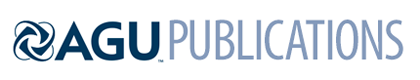


*Geophysical Research Letters*

Supporting Information for

Joint CO2 and COS flux measurements reveal systematic underestimation of gross primary productivity across major biomes

F. M. Spielmann^1^, G. Wohlfahrt^1^, A. Hammerle^1^, F. Kitz^1^, M. Migliavacca^2^, G. Alberti^3,4^, A. Ibrom^5^, T. S. El-Madany^2^, K. Gerdel^1^, G. Moreno^6^, O. Kolle^2^, T. Karl^7^, A. Peressoti^3^, G. Delle Vedove^3^

^1^ Department of Ecology, University of Innsbruck, 6020 Innsbruck, AUSTRIA

^2^ Department of Agricultural, Food, Environmental and Animal Sciences, University of Udine, 33100 Udine, Italy

^3^ Department of Environmental Engineering, Technical University of Denmark, 2800 Kongens Lyngby, Denmark

^4^ Institute of Atmospheric and Cryospheric Sciences, University of Innsbruck, 6020 Innsbruck, AUSTRIA

^5^ Department of Biogeochemical Integration, Max Planck Institute for Biogeochemistry, 07745 Jena, Germany

^6^ INDEHESA-Forest Research Group, Universidad de Extremadura, 10600 Plasencia, Spain

^7^ CNR-IBIMET, via Caproni 8, Firenze, Italy

**Contents of this file**

Text S1 to S4

Figures S1 to S20

Tables S1 to S4

**Introduction**

Text S1 Supplementary information on the measurement campaigns

Due to the type of management i.e. the cutting and removal of the grass up to 4 times a year, for the comparison with the other sites, we selected time periods at GRA before the cuts between June and August, having an LAI between 4 and 6 m^2^ m^-2^. The campaign at (SAV) happened at the end of the growing season (April-May 2016), before going into senescence after our campaign due to the summer drought. Measurements at (DBF) were made in the middle of the growing season. We started our measurements at (CRO) 35 days after the seeds were planted.

Text S2 Supplementary information on mole fraction measurements

To correct for the laser drift (Kooijmans et al., 2016), we linearly interpolated in time between the obtained calibration values and subtracted the retrieved values from the high frequency data. A sample of the calculated calibration offsets can be seen in Fig. S1.

Besides of the correct COS ambient mole fractions, the LRU (Eq. 1) also depends on the correct retrieval of CO_2_ mole fractions, which were measured using the on-site infrared gas analyzers (IRGA) (Table S1). We used the resulting half hourly means of both COS and CO_2_ mole fractions to investigate how their ratio differs among sites and changes over the course of the day (Fig. S2). We found that GRA and SAV had ratios of about 0.8 ppm ppt^-1^ during daytime hours, whereas we observed DBF and CRO had midday ratios of around 0.9 ppm ppt^–1^, which lie at the upper end of the values obtained in other studies (see Table S2). During nighttime, the ratios increased for GRA, SAV and CRO, due to ecosystem respiration and the continuous uptake of COS from soils and/or plants. Missing absolute COS values, due to issues with the calibration gas cylinders were gap filled in DBF using the mean half hourly diel time series of the COS mole fractions of the remaining campaign duration. The importance of accurately quantifying correct ambient mole fractions can be illustrated by assuming a hypothetical CO_2_ mole fraction of 400 ppm and a COS mole fraction of 500 ppt and CO_2_ (20 µmol m^-2^ s^-1^) and COS (40 pmol m^-2^ s^-1^) fluxes (measured maximum midday fluxes at GRA). Inserting these values into Eq. S1, LRU would change from 2 to 1.33 for an offset correction of ± 100 ppt COS (Figure S1).

$LRU = \frac{F_{COS}/\chi_{COS}}{F_{CO2}/\chi_{CO2}}$ (S1)

Text S3 Supplementary information on soil models

On the basis of the measured soil fluxes and additionally retrieved meteorological and soil data (incident shortwave radiation reaching the soil surface, soil moisture and soil temperature), a random forest regression model (Liaw & Wiener, 2001) was trained for each site using the scikit-learn (sklearn Ver. 0.19.1) package, the pandas library and the Python Software Distribution Anaconda (Ver. 5.2.0) in the command shell Ipython (Ver. 6.4.0) based on the Programming language Python (Ver. 3.3.5), to estimate the soil fluxes for the whole duration of the measurement campaigns on a half-hourly basis. As vegetation had to be removed at SAV and GRA in order to be able to access the soil surface, radiation incident at the soil surface under undisturbed conditions was modeled at these sites using Beer-Lambert law calibrated against in situ soil radiation measurements. We used a dynamic GAI for GRA as the site was cut once during the two month period of interest, whereas we used a fixed LAI in SAV, which was constantly grazed by cattle. The LAI of the trees was projected over the whole ground area.

A random forest with 10000 trees was grown which resulted in an out of bag (OOB) score of 0.794. The optimal input parameters, including maximum tree depth, were determined with the function GridSearchCV from the sklearn package. The relative importance of the variables resulting from the random forest regression including the measurements of all sites showed that the most important variable was the light reaching the soil surface, which accounted for more than 70 % of the total variance explained by the random-forest model (see Fig. S3).

Text S4.Supplementary information on the Bayesian model inversion

The Bayesian model inversion scheme DREAM avoids convergence problems by (i) running several chains (10 in our case) simultaneously learning from each other to achieve global parameter space extrapolation, (ii) tuning scale and orientation of the proposal distribution during parameter search and (iii) applying an outlier chain handling (Vrugt & Ter Braak, 2011). In addition to parameter estimates, the inversion scheme provides quantitative measures of uncertainty (parameter and predictive uncertainty) and correlation among parameters (Van Oijen et al., 2005).

While an informative prior distribution was used for the parameter ι retrieved from LRU values described in literature, non-informative (uniform distribution) priors were used for the remaining parameters to be estimated. The bounds used for all parameters in our models are reported in Table S3. Model calibration was performed by maximizing the negative logarithm of a likelihood function, quantifying the probability of observed data being generated by a particular parameter set in combination with the given model (Schoups & Vrugt, 2010). Because of the possibility of non-normality, heteroscedasticity and correlation of model residuals we applied a generalized likelihood function (Schoups & Vrugt, 2010) for both constraints (i.e. FCOS and NEE). These error distributions do account for measurement, model input and model structural errors in a lumped manner (Schoups & Vrugt, 2010). Using a Markov Chain Monte Carlo algorithm representative samples were drawn from the multi-dimensional parameter space to approximate posterior parameter probability distributions (Van Oijen et al., 2005). Posterior parameter distributions, residual error distributions and consequently parameter- and model predictive uncertainties (see Fig. S3 & Fig. S18) were calculated from the last 2950 parameter sets from the posterior distribution after reaching convergence (Gelman-Rubin statistics < 1.2 (A. Gelman, 1996)) between the 10 chains run in parallel. Both models (i.e. FP and FP+) were based on the same dataset as we filtered for times when only both constraints (i.e. FCOS and NEE) were available. We optimized the sum of the generalized likelihood function for both constraints within the FP+ model in parallel and equally weighted.

GPP simulated with FP and FP+ models was compared on a relative (percentage) basis in order to control for differences in GPP between sites (Fig. 3) by calculating the daily sums of GPP, forming the percentage difference between FP and FP+ models on a daily basis and then calculating the average and standard deviation over the duration of the measurement campaigns.


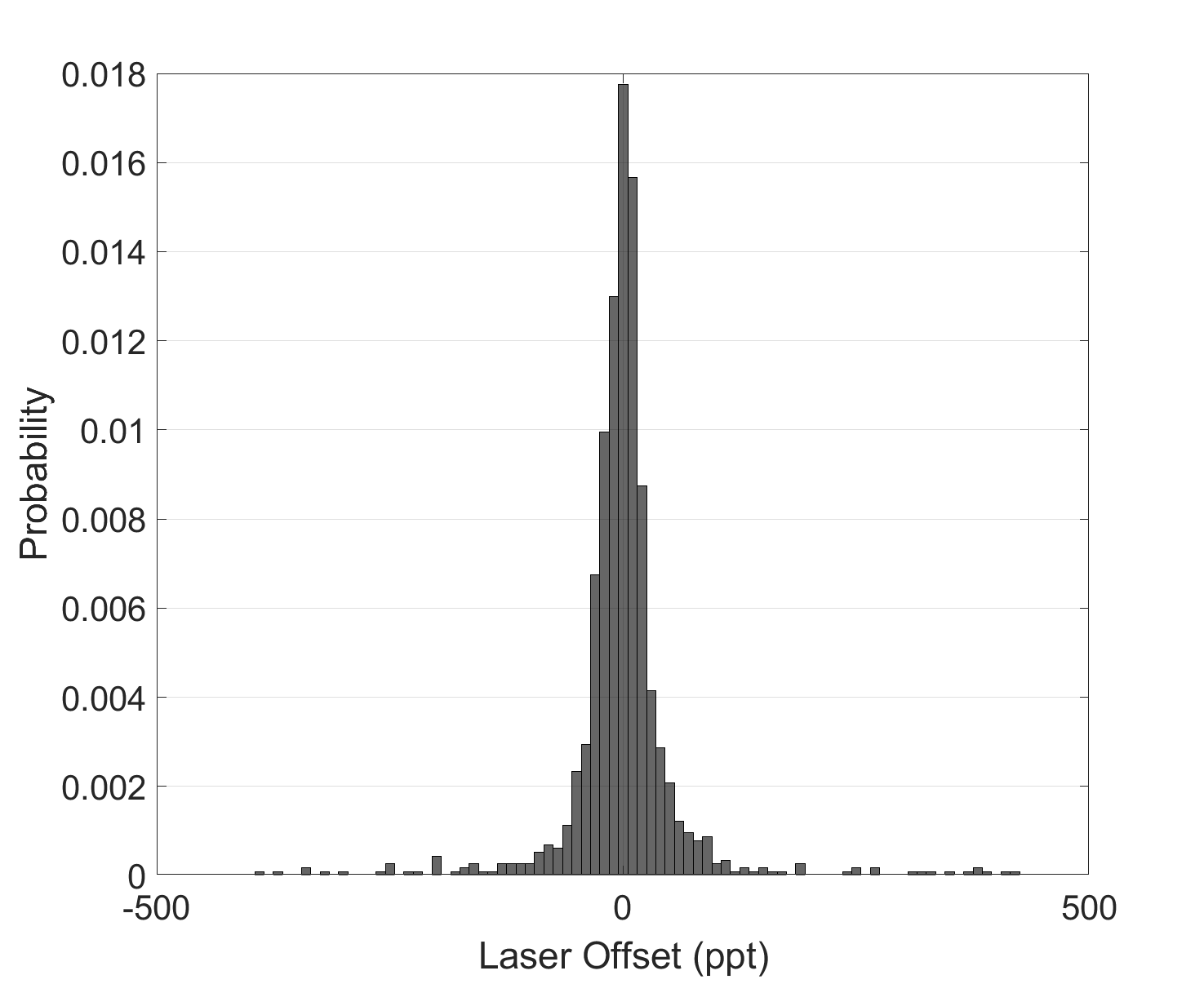
**Figure S1. Histogram of COS offset.** Probability density function of the half hourly laser offset for COS (ppt) over the course of the measurement campaign at the field site SAV.


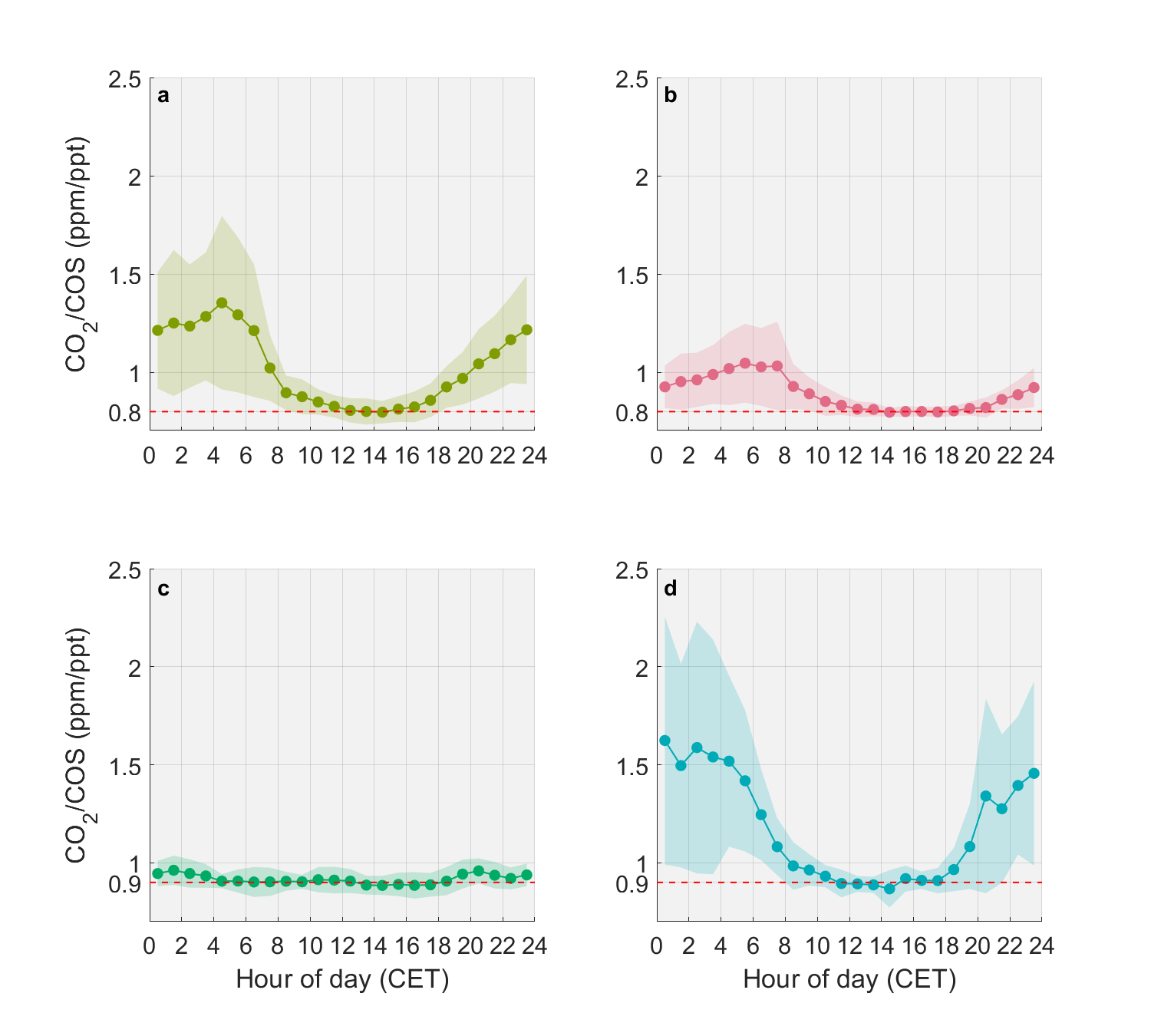
Figure S2. Mean diel variation of the CO2 to COS concentration ratio. plotted for (a) GRA (b) SAV (c) DBF and (d) CRO. Solid circles indicate the hourly mean, the shaded area represents ± one standard deviation of the mean, the dashed line represents the value of 0.8 (ppm/ppt) in a) and b) and 0.9 (ppm/ppt) in (c) and (d).


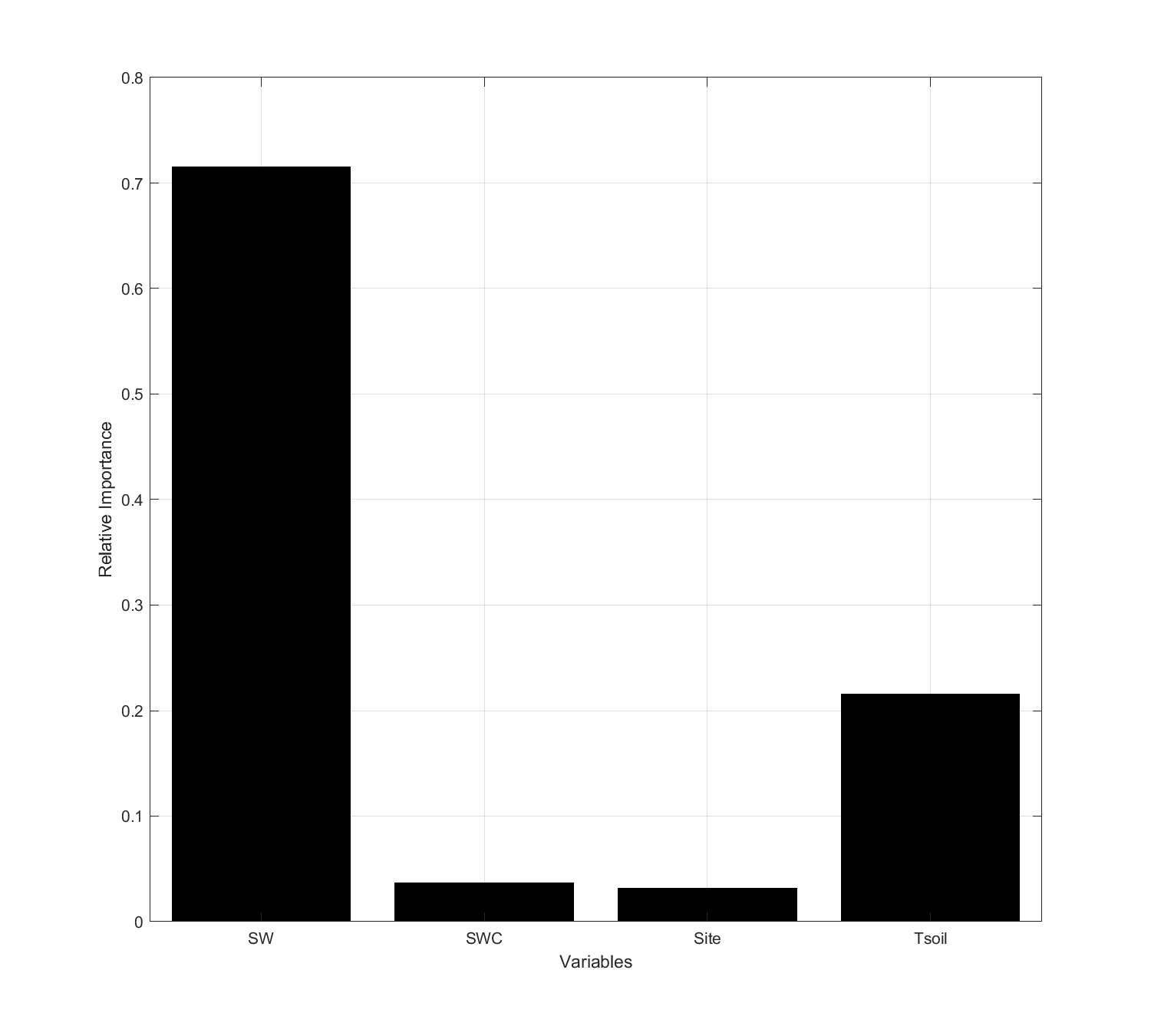
Figure S3. Relative importance of the soil model input variables. Relative importance of the incoming shortwave radiation (SW), soil water content (SWC), site and the soil temperature at 5cm depth (Tsoil) resulting from the random forest regression model including the soil chamber measurements of all sites.


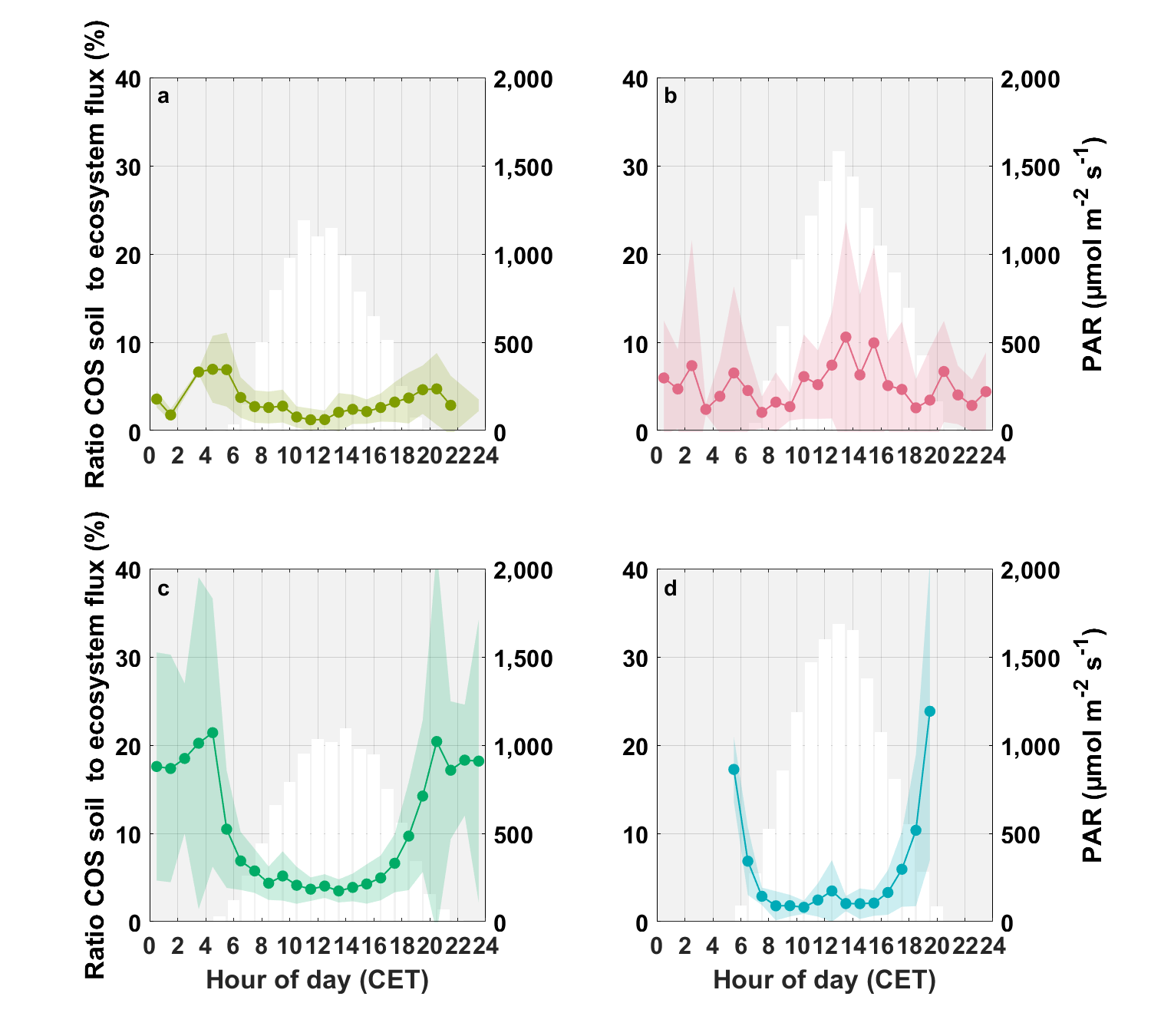
Figure S4. COS soil to ecosystem flux ratios. Mean diel variation of the ratio between the absolute COS soil to ecosystem flux on the left y axis for (a) GRA (b) SAV (c) DBF and (d) CRO. Ecosystem fluxes are retrieved from eddy covariance measurements whereas the soil fluxes were modeled (see Materials and Methods – Soil models). Filled circles represent the hourly mean, shaded areas represent ± one standard deviation of the mean. The photosynthetic active radiation is plotted as half hourly means on the right y axis of each plot as bar graph.


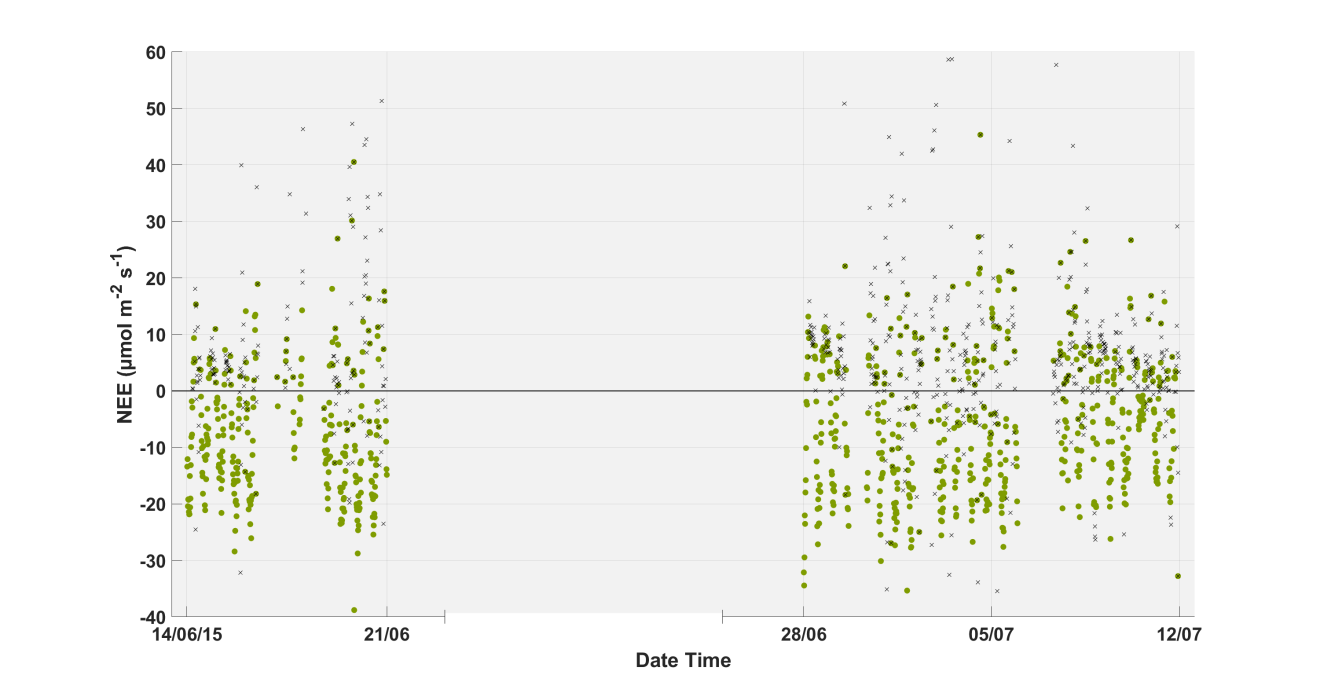
Figure S5. NEE time series for GRA. Black x’s indicate values below the limit of detection (Langford et al., 2015), which cannot be distinguished from zero fluxes.


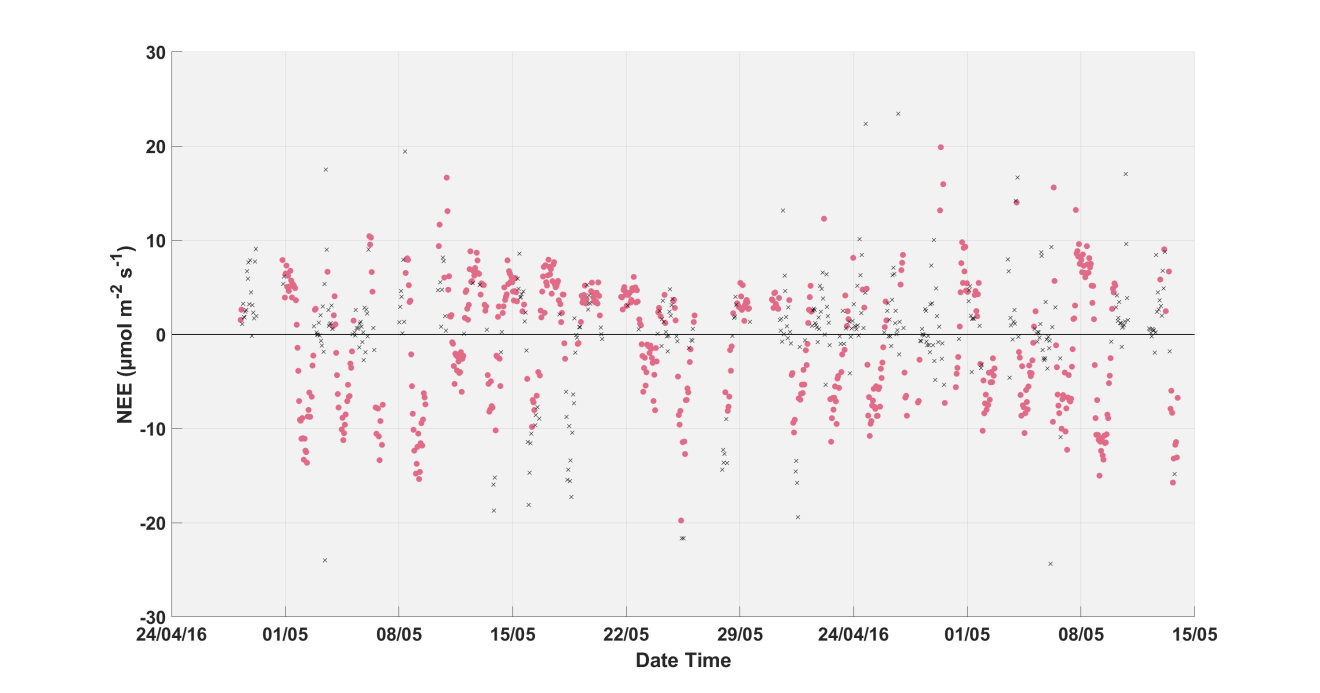
Figure S6. NEE time series for SAV. Black x’s indicate values below the limit of detection (Langford et al., 2015), which cannot be distinguished from zero fluxes.


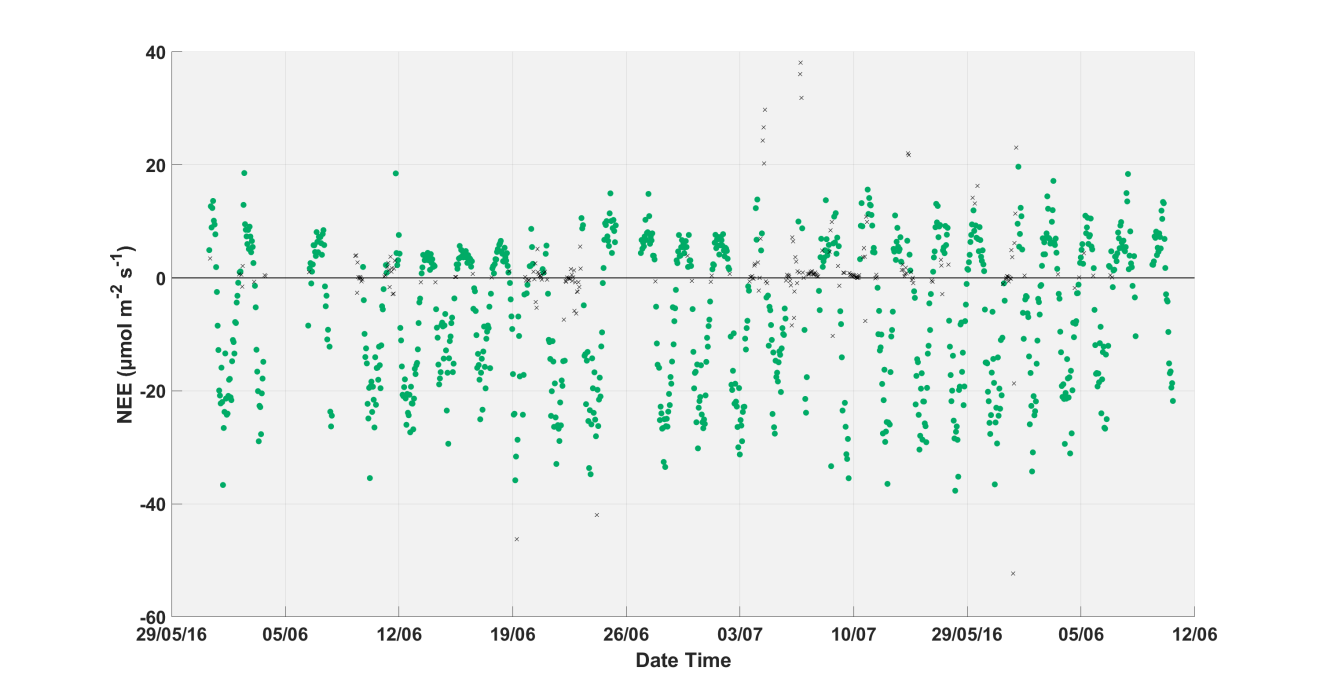
Figure S7. NEE time series for DBF. Black x’s indicate values below the limit of detection (Langford et al., 2015), which cannot be distinguished from zero fluxes.


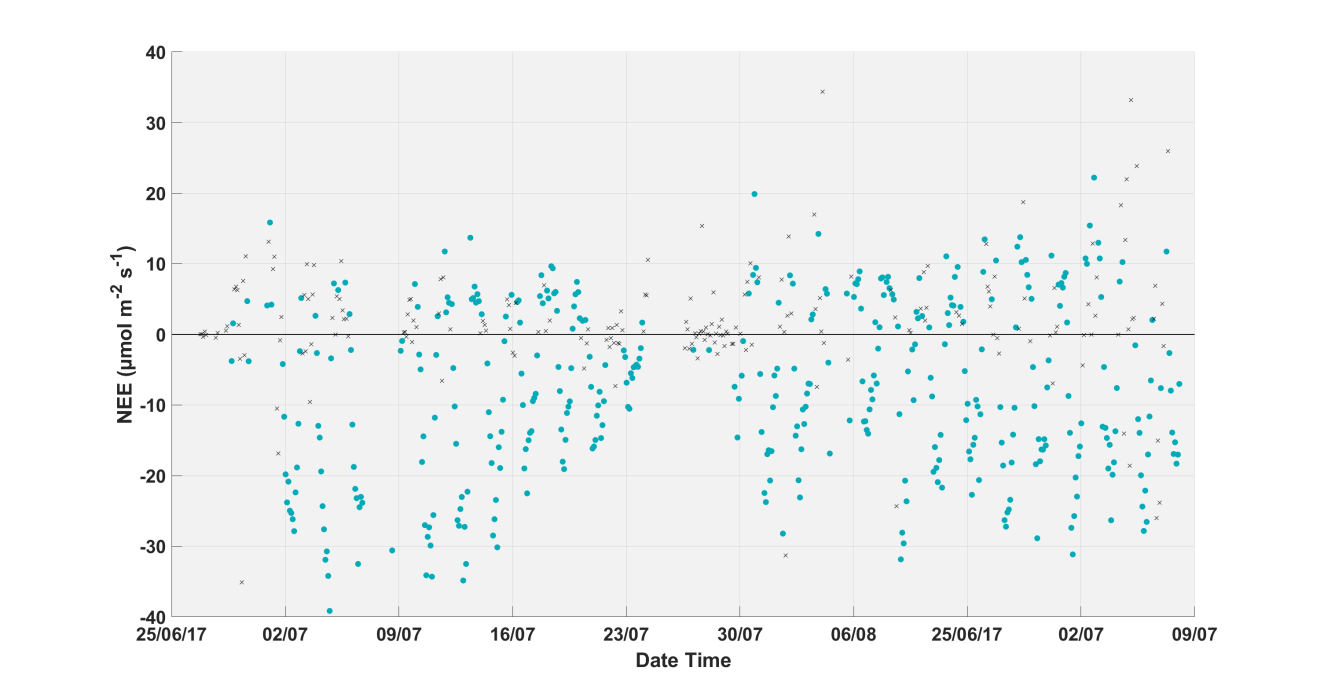
Figure S8. NEE time series for CRO. Black x’s indicate values below the limit of detection (Langford et al., 2015), which cannot be distinguished from zero fluxes.


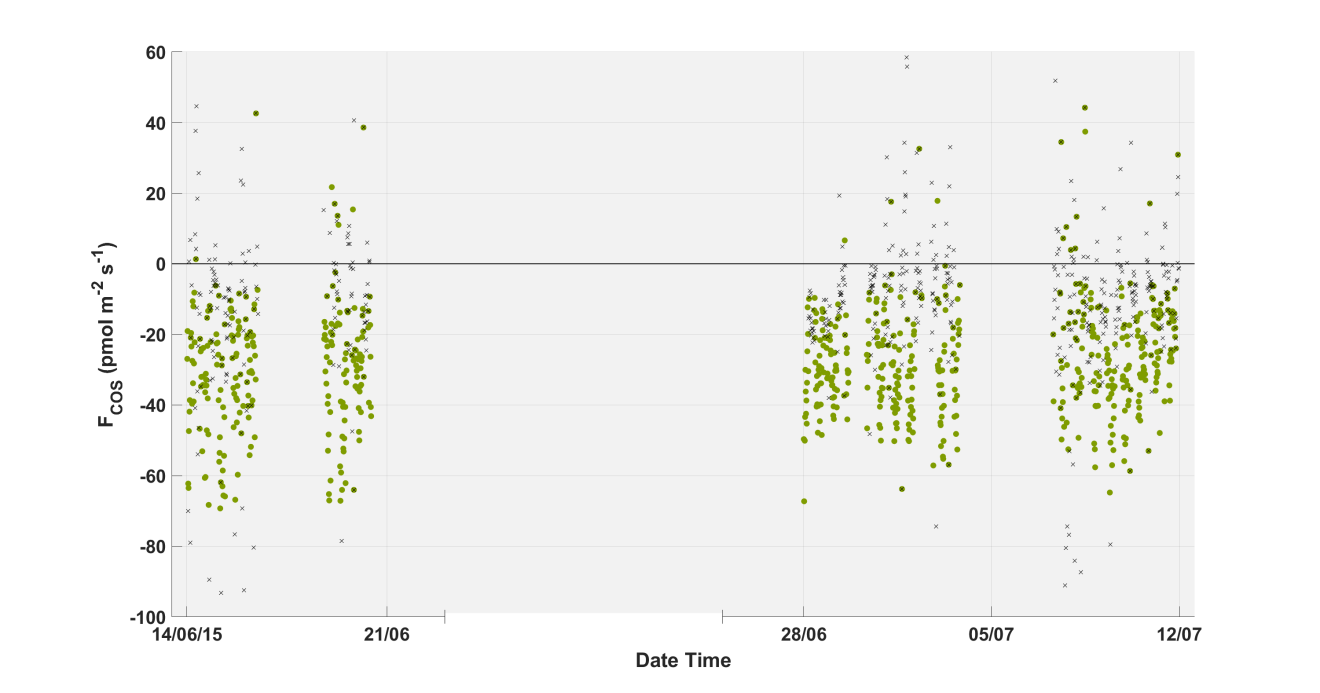
Figure S9. COS ecosystem flux time series for GRA. Black x’s indicate values below the limit of detection (Langford et al., 2015), which cannot be distinguished from zero fluxes.


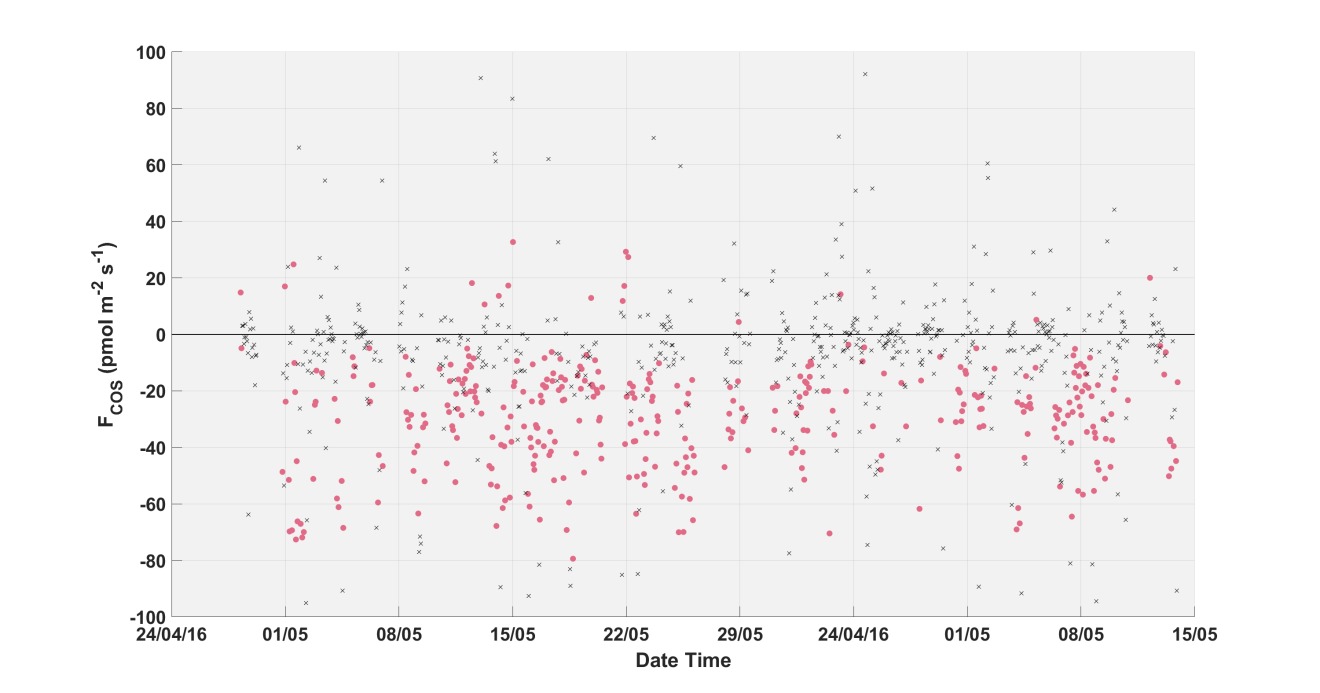
Figure S10. COS ecosystem flux time series for SAV. Black x’s indicate values below the limit of detection (Langford et al., 2015), which cannot be distinguished from zero fluxes.


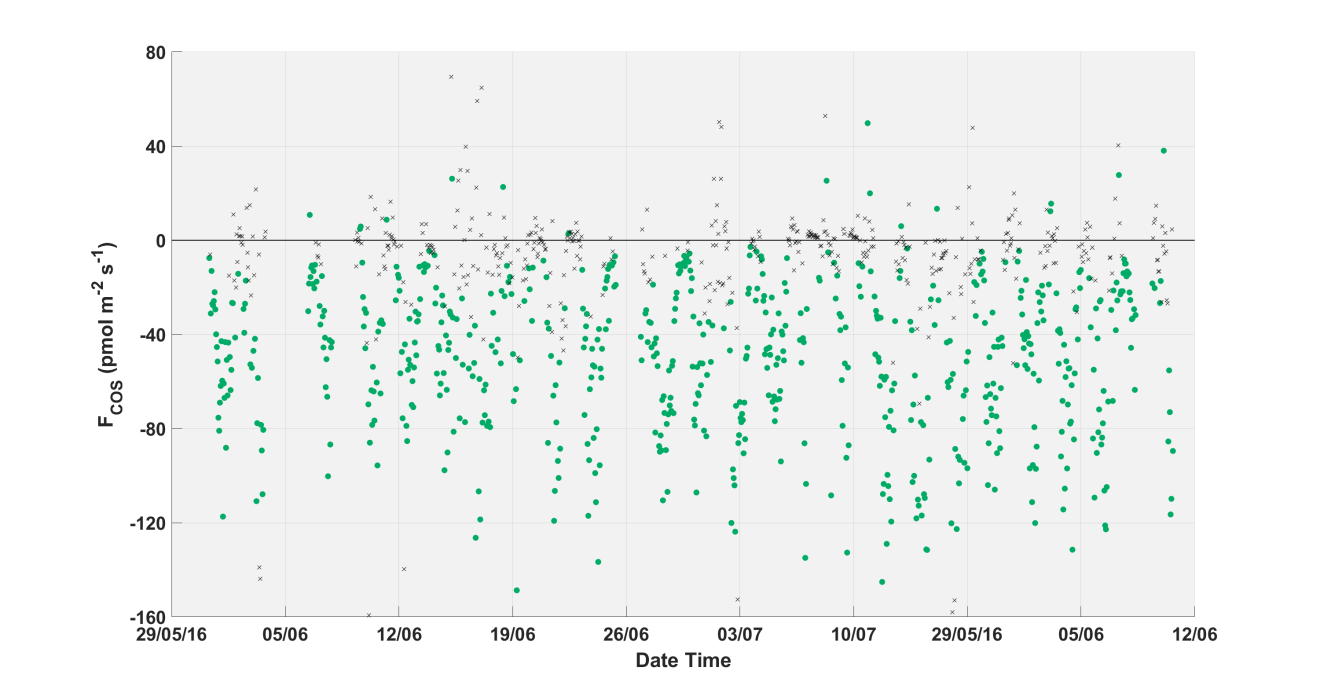
Figure S11. COS ecosystem flux time series for DBF. Black x’s indicate values below the limit of detection (Langford et al., 2015), which cannot be distinguished from zero fluxes.


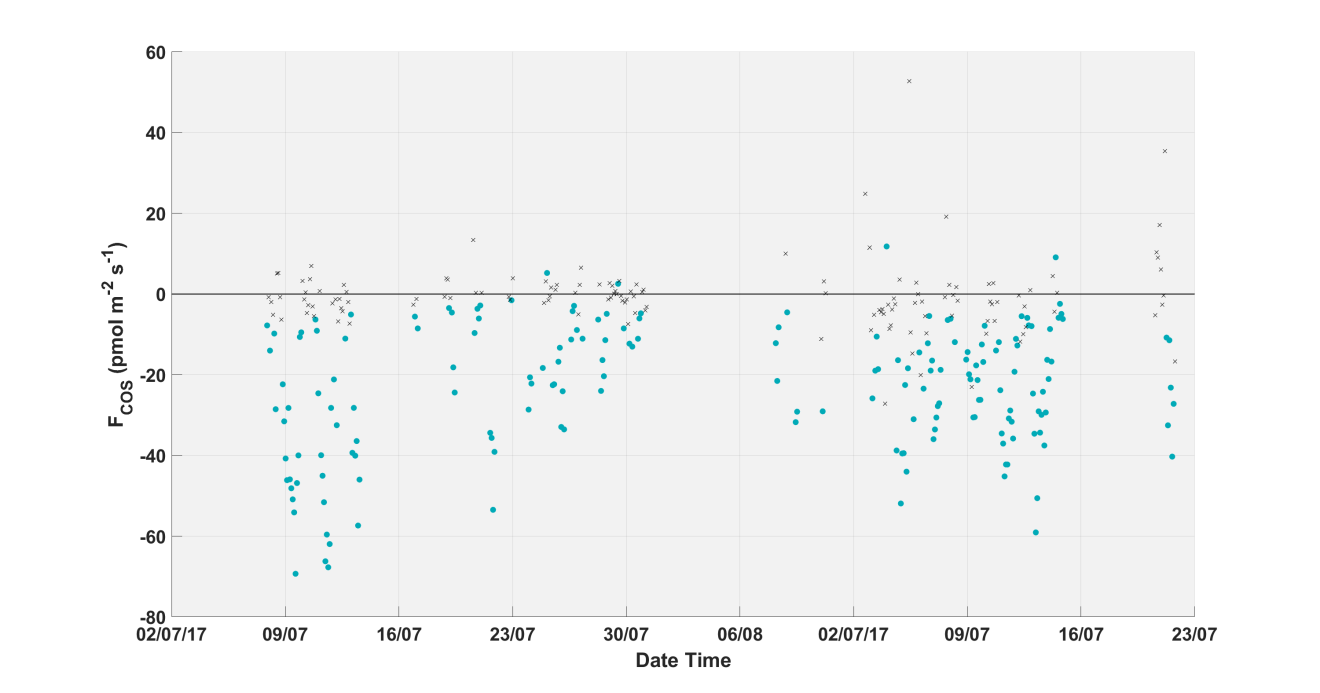
Figure S12. COS ecosystem flux time series for CRO. Black x’s indicate values below the limit of detection (Langford et al., 2015), which cannot be distinguished from zero fluxes.


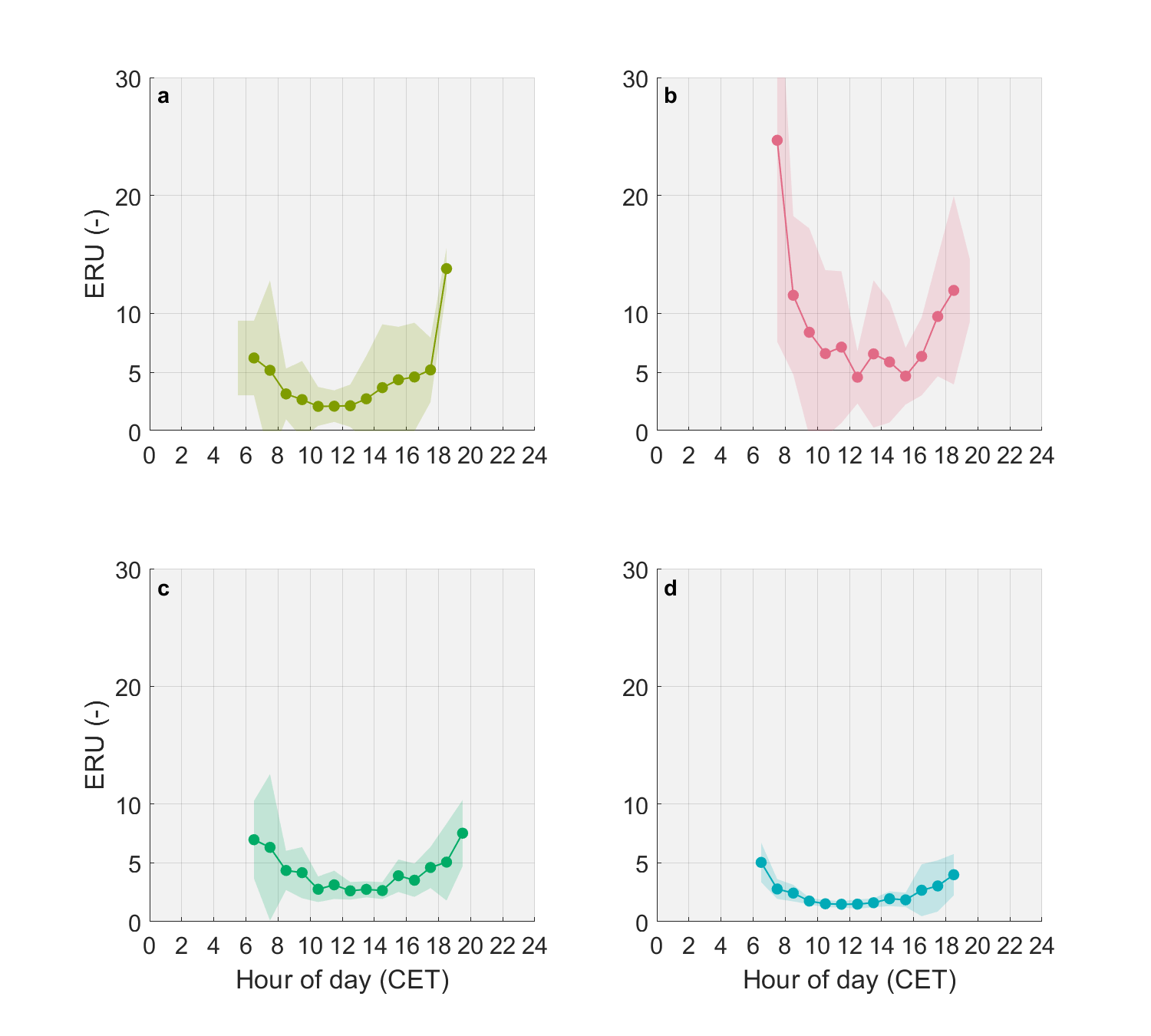
Figure S13. Mean diel variation of ERU. Mean diel variation of the ratio between the COS ecosystem flux and NEE normalized by their corresponding ambient concentrations (ERU) for (a) GRA (b) SAV (c) DBF and (d) CRO


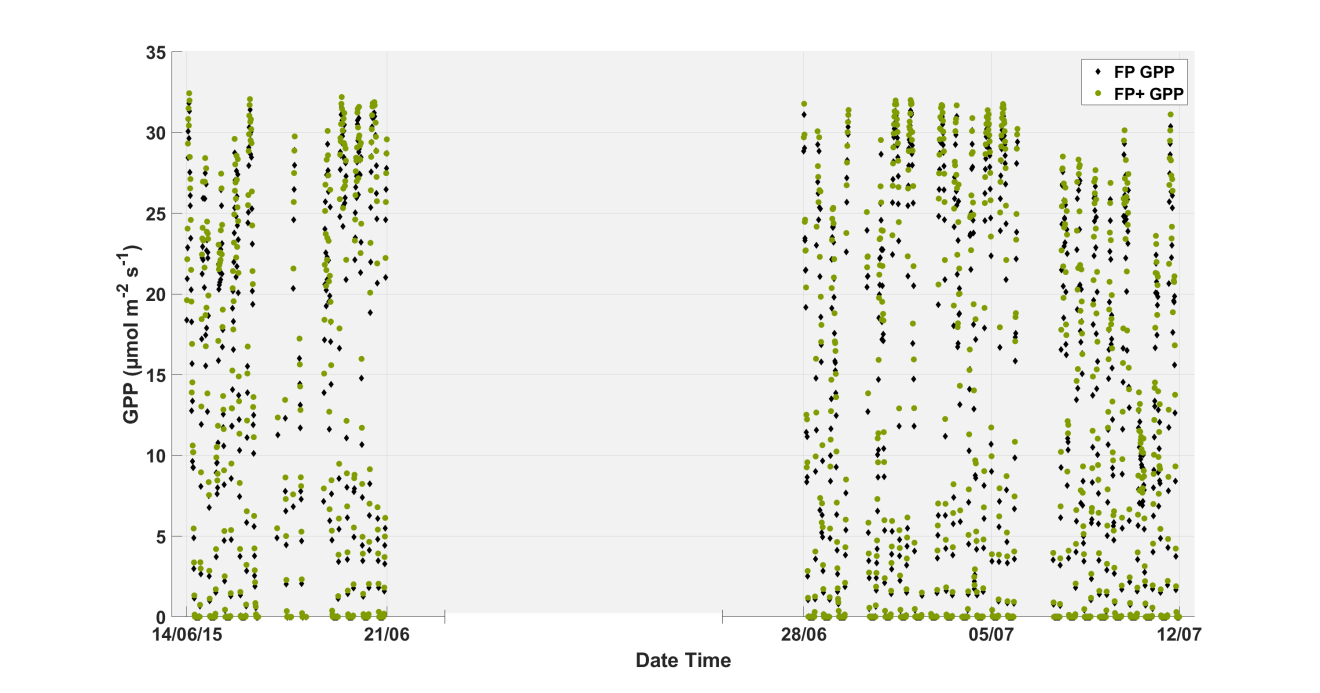
Figure S14. GPP time series for GRA. Black x’s indicate values below the limit of detection (Langford et al., 2015), which cannot be distinguished from zero fluxes.


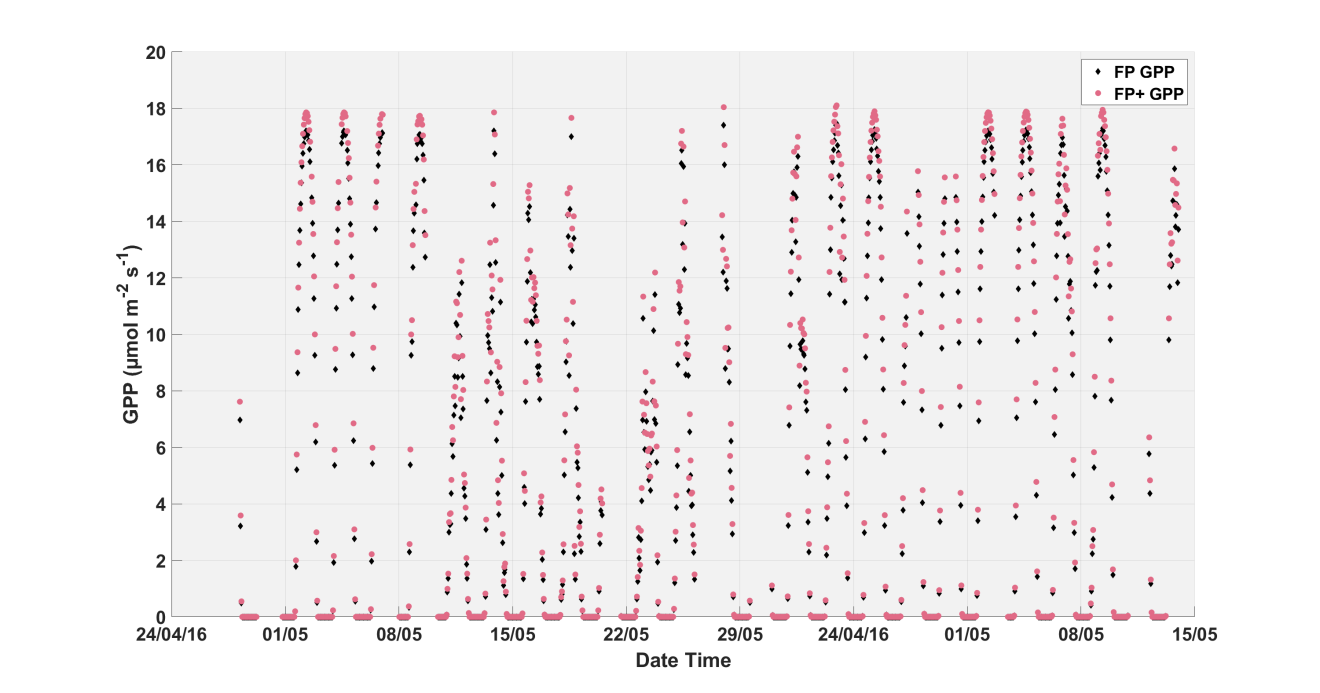
Figure S15. GPP time series for SAV. Black x’s indicate values below the limit of detection (Langford et al., 2015), which cannot be distinguished from zero fluxes.


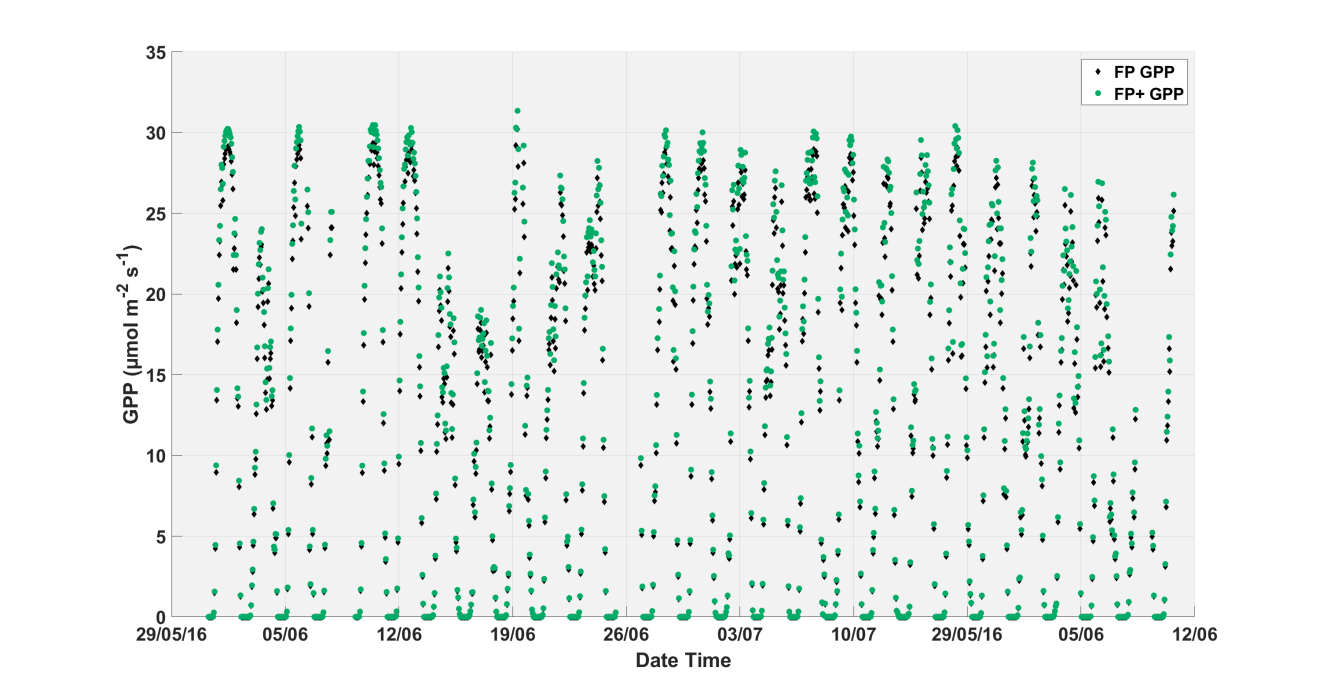
Figure S16. GPP time series for DBF. Black x’s indicate values below the limit of detection (Langford et al., 2015), which cannot be distinguished from zero fluxes.


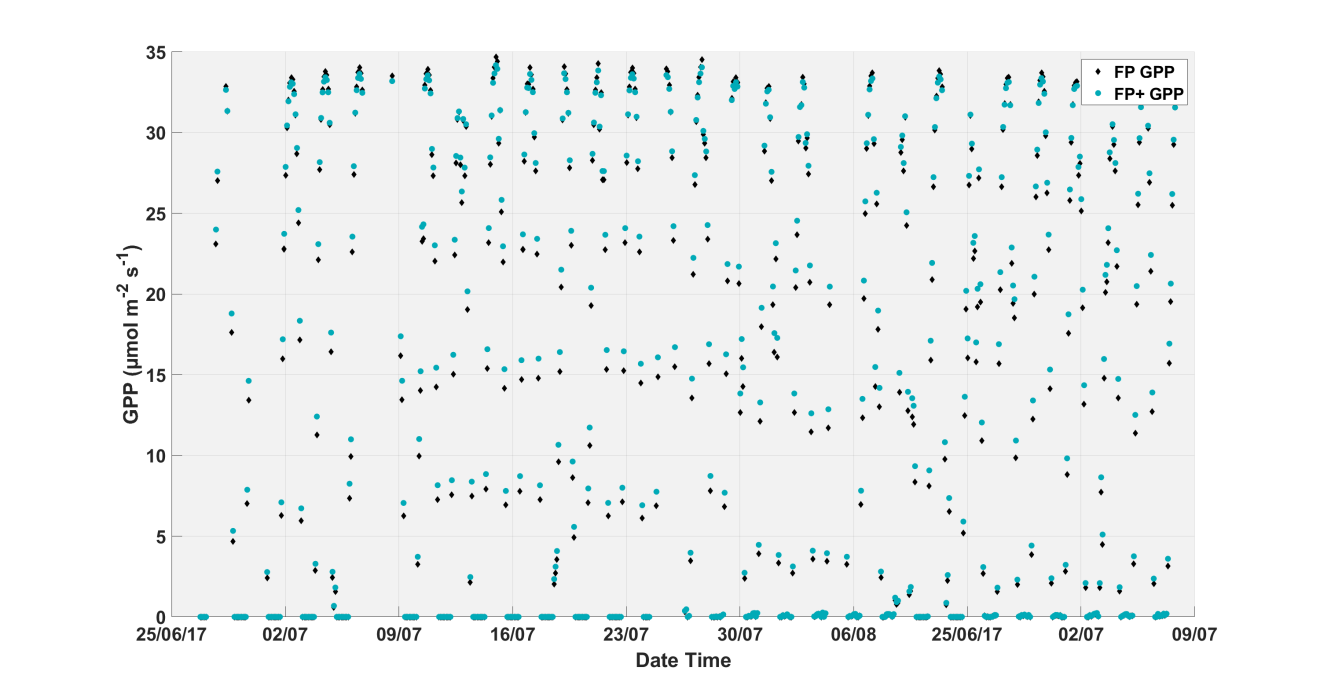
Figure S17. GPP time series for CRO. Black x’s indicate values below the limit of detection (Langford et al., 2015), which cannot be distinguished from zero fluxes.


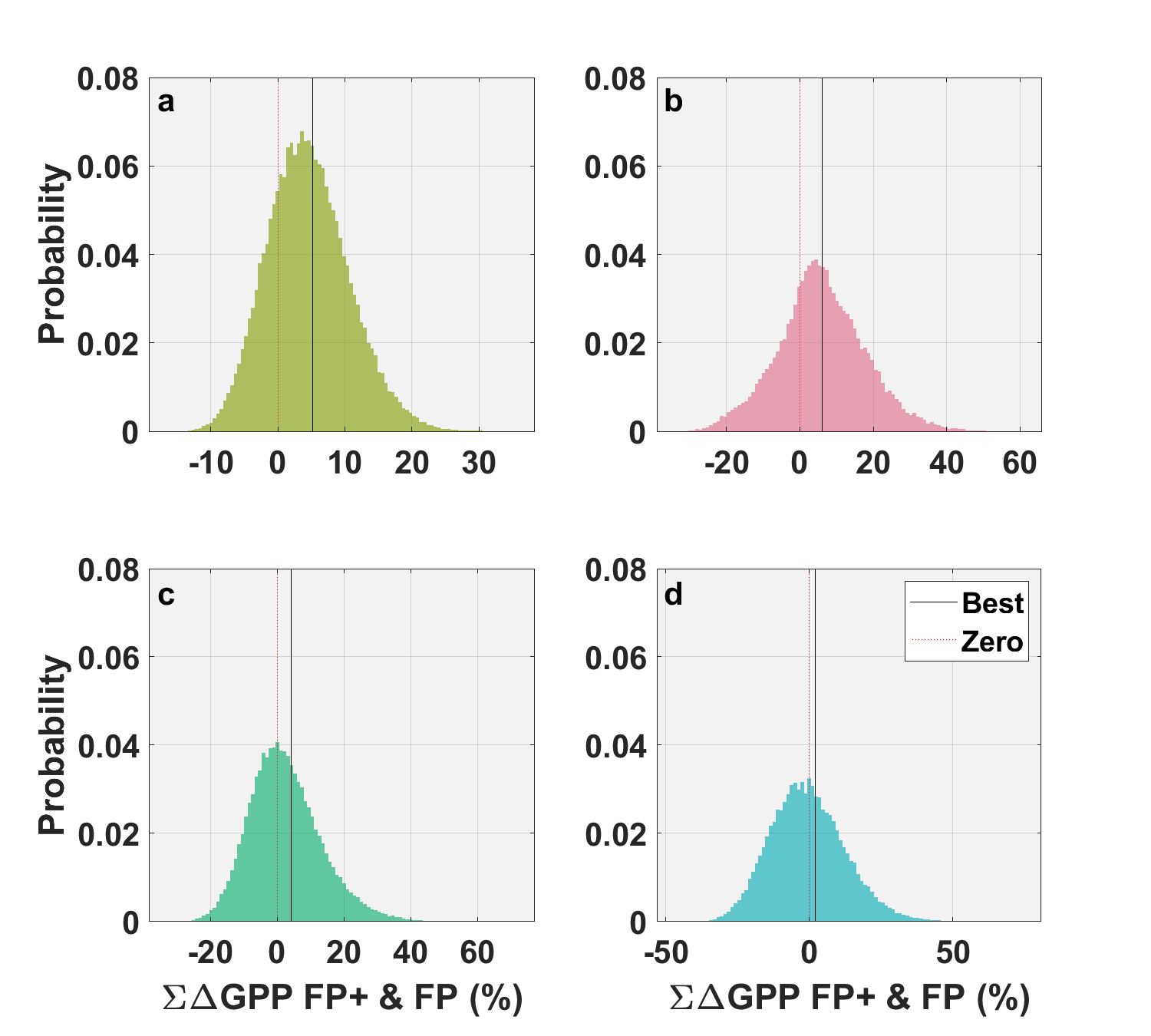
Figure S18. Probability density function of the difference in cumulative GPP over the course of the campaigns between the last 2950 outputs of the FP+ and FP model after reaching convergence for (a) GRA (b) SAV (c) DBF and (d) CRO in percent. Days with missing values were excluded from this analysis. Positive values indicate higher GPP resulting from the FP+ model. The dashed red line represents the zero line. The black solid line represents difference of the model outputs obtained with the best parameter sets.


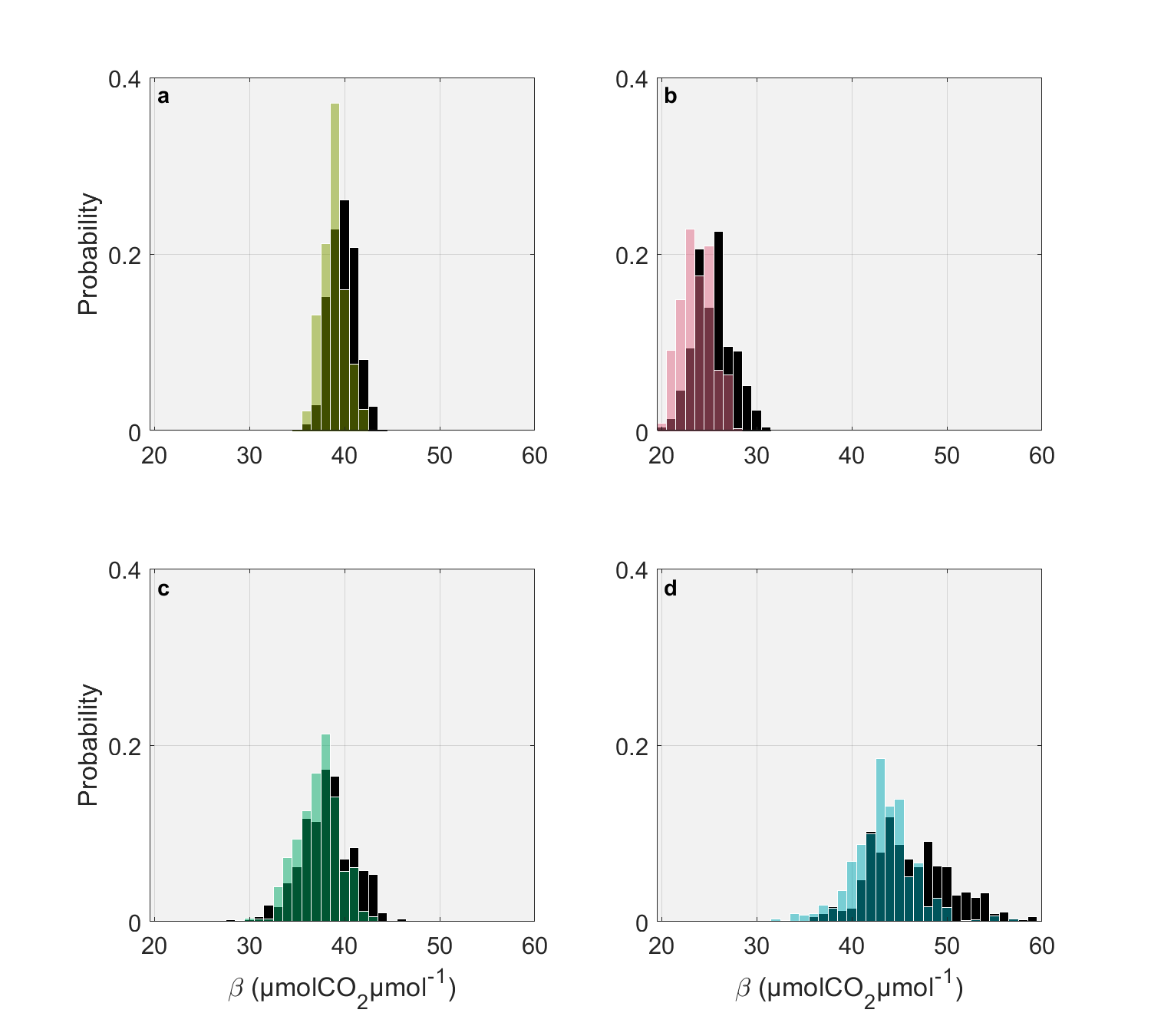
Figure S19. Model parameter output of β. Histogram of the distribution of the maximum CO2 uptake rate of the canopy at light saturation for the FP model (black bars) and the FP+ model (colored bars) for (a) GRA (b) SAV (c) DBF and (d) CRO.


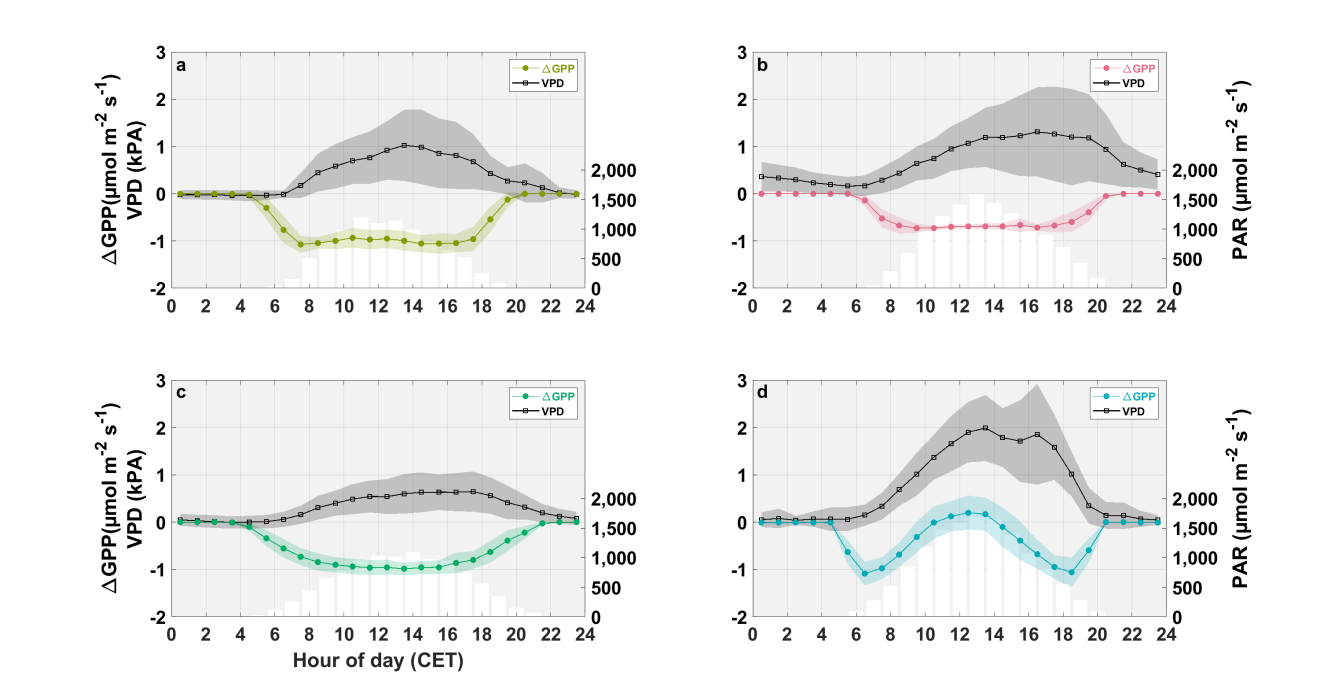
Figure S20. Mean diel variation of the GPP difference resulting from the FP and FP+ model for (a) GRA (b) SAV (c) DBF and (d) CRO. Filled circles represent the hourly means of the difference in GPP, while empty squares depict the hourly means of the VPD. Negative values indicate higher GPP resulting from the FP+ model. Shaded areas represent ± one standard deviation of the mean. The photosynthetic active radiation is plotted as half hourly means on the right y axis of each plot as a bar graph.

Table S1. Site description (Braendholt et al., 2018; El-Madany et al., 2018; Hortnagl et al., 2011; Hortnagl & Wohlfahrt, 2014)

Table S2. Midday concentration ratios retrieved from (Asaf et al., 2013; Berkelhammer et al., 2014; Campbell et al., 2017)

Table S3. Model parameter bounds: Lower and upper bounds for each parameter used in the FP and FP+ model for the estimation procedure with DREAM.

Table S4 Best parameter set output for model parameter ι

References

A. Gelman, G. O. R., W. R. Gilks. (1996). Efficient Metropolis jumping rules. In J. O. B. J. M. Bernardo, A. P. Dawid, and A. F. M. Smith (Ed.), *Bayesian statistics 5* (Vol. 5, pp. 42). Oxford University Press.

Asaf, D., Rotenberg, E., Tatarinov, F., Dicken, U., Montzka, S. A., & Yakir, D. (2013). Ecosystem photosynthesis inferred from measurements of carbonyl sulphide flux. *Nature Geoscience, 6*(3), 186-190. <Go to ISI>://WOS:000316945800017

Berkelhammer, M., Asaf, D., Still, C., Montzka, S., Noone, D., Gupta, M., et al. (2014). Constraining surface carbon fluxes using in situ measurements of carbonyl sulfide and carbon dioxide. *Global Biogeochemical Cycles, 28*(2), 161-179. <Go to ISI>://WOS:000333014200007

Braendholt, A., Ibrom, A., Larsen, K. S., & Pilegaard, K. (2018). Partitioning of ecosystem respiration in a beech forest. *Agricultural and Forest Meteorology, 252*, 88-+. Article. <Go to ISI>://WOS:000427341400009

<https://www.sciencedirect.com/science/article/pii/S0168192318300121?via%3Dihub>

Campbell, J. E., Whelan, M. E., Berry, J. A., Hilton, T. W., Zumkehr, A., Stinecipher, J., et al. (2017). Plant Uptake of Atmospheric Carbonyl Sulfide in Coast Redwood Forests. *Journal of Geophysical Research-Biogeosciences, 122*(12), 3391-3404. <Go to ISI>://WOS:000423221300018

<https://agupubs.onlinelibrary.wiley.com/doi/pdf/10.1002/2016JG003703>

El-Madany, T. S., Reichstein, M., Perez-Priego, O., Carrara, A., Moreno, G., Pilar Martín, M., et al. (2018). Drivers of spatio-temporal variability of carbon dioxide and energy fluxes in a Mediterranean savanna ecosystem. *Agricultural and Forest Meteorology, 262*, 258-278. <http://www.sciencedirect.com/science/article/pii/S0168192318302314>

Hortnagl, L., Bamberger, I., Graus, M., Ruuskanen, T. M., Schnitzhofer, R., Muller, M., et al. (2011). Biotic, abiotic and management controls on methanol exchange above a temperate mountain grassland. *J Geophys Res Biogeosci, 116*(G3), G03021-G03021. <https://www.ncbi.nlm.nih.gov/pubmed/24349901>

Hortnagl, L., & Wohlfahrt, G. (2014). Methane and nitrous oxide exchange over a managed hay meadow. *Biogeosciences, 11*(24), 7219-7236. Article. <Go to ISI>://WOS:000347959800004

<https://www.ncbi.nlm.nih.gov/pmc/articles/PMC4373549/pdf/emss-62480.pdf>

Kooijmans, L. M. J., Uitslag, N. A. M., Zahniser, M. S., Nelson, D. D., Montzka, S. A., & Chen, H. L. (2016). Continuous and high-precision atmospheric concentration measurements of COS, CO2, CO and H2O using a quantum cascade laser spectrometer (QCLS). *Atmospheric Measurement Techniques, 9*(11), 5293-5314. <Go to ISI>://WOS:000387116100002

<http://www.atmos-meas-tech.net/9/5293/2016/amt-9-5293-2016.pdf>

Langford, B., Acton, W., Ammann, C., Valach, A., & Nemitz, E. (2015). Eddy-covariance data with low signal-to-noise ratio: time-lag determination, uncertainties and limit of detection. *Atmospheric Measurement Techniques, 8*(10), 4197-4213. Article. <Go to ISI>://WOS:000364317600015

<http://www.atmos-meas-tech.net/8/4197/2015/amt-8-4197-2015.pdf>

Liaw, A., & Wiener, M. (2001). *Classification and Regression by RandomForest* (Vol. 23).

Schoups, G., & Vrugt, J. A. (2010). A formal likelihood function for parameter and predictive inference of hydrologic models with correlated, heteroscedastic, and non-Gaussian errors. *Water Resources Research, 46*, 17. Article. <Go to ISI>://WOS:000283551300002

<http://onlinelibrary.wiley.com/store/10.1029/2009WR008933/asset/wrcr12578.pdf?v=1&t=jesgxq4c&s=54fa4d90a5a1b1ecfc36b0993f2f99b553b5a1aa>

Van Oijen, M., Rougier, J., & Smith, R. (2005). Bayesian calibration of process-based forest models: bridging the gap between models and data. *Tree Physiology, 25*(7), 915-927. <Go to ISI>://WOS:000230164300013

Vrugt, J. A., & Ter Braak, C. J. F. (2011). DREAM((D)): an adaptive Markov Chain Monte Carlo simulation algorithm to solve discrete, noncontinuous, and combinatorial posterior parameter estimation problems. *Hydrology and Earth System Sciences, 15*(12), 3701-3713. <Go to ISI>://WOS:000298366200007

<https://www.hydrol-earth-syst-sci.net/15/3701/2011/hess-15-3701-2011.pdf>
